# The genome sequence of Sea-Island cotton (*Gossypium barbadense*) provides insights into the allopolyploidization and development of superior spinnable fibres

**DOI:** 10.1038/srep17662

**Published:** 2015-12-04

**Authors:** Daojun Yuan, Zhonghui Tang, Maojun Wang, Wenhui Gao, Lili Tu, Xin Jin, Lingling Chen, Yonghui He, Lin Zhang, Longfu Zhu, Yang Li, Qiqi Liang, Zhongxu Lin, Xiyan Yang, Nian Liu, Shuangxia Jin, Yang Lei, Yuanhao Ding, Guoliang Li, Xiaoan Ruan, Yijun Ruan, Xianlong Zhang

**Affiliations:** 1 National Key Laboratory of Crop Genetic Improvement, Huazhong Agricultural University, Shizishan Street, Wuhan, Hubei 430070, China; 2 The Jackson Laboratory for Genome Medicine, 10 Discovery Drive, Farmington, CT 06032, USA; 3 College of Informatics, Huazhong Agricultural University, Shizishan Street, Wuhan, Hubei 430070, China; 4 Department of Genetics and Developmental Biology, University of Connecticut Health Center, 400 Farmington Ave, Farmington, CT 06032, USA

## Abstract

*Gossypium hirsutum* contributes the most production of cotton fibre, but *G. barbadense* is valued for its better comprehensive resistance and superior fibre properties. However, the allotetraploid genome of *G. barbadense* has not been comprehensively analysed. Here we present a high-quality assembly of the 2.57 gigabase genome of *G. barbadense*, including 80,876 protein-coding genes. The double-sized genome of the A (or At) (1.50 Gb) against D (or Dt) (853 Mb) primarily resulted from the expansion of Gypsy elements, including Peabody and Retrosat2 subclades in the Del clade, and the Athila subclade in the Athila/Tat clade. Substantial gene expansion and contraction were observed and rich homoeologous gene pairs with biased expression patterns were identified, suggesting abundant gene sub-functionalization occurred by allopolyploidization. More specifically, the *CesA* gene family has adapted differentially temporal expression patterns, suggesting an integrated regulatory mechanism of *CesA* genes from At and Dt subgenomes for the primary and secondary cellulose biosynthesis of cotton fibre in a “relay race”-like fashion. We anticipate that the *G. barbadense* genome sequence will advance our understanding the mechanism of genome polyploidization and underpin genome-wide comparison research in this genus.

Cotton has been cultivated for its fibre for more than 7,000 years. Despite the availability of the petroleum-derived synthetic fibre alternatives, cotton fibre continues to serve as the world’s most important natural renewable sources for textiles. Cotton is grown in more than 80 countries; its production provides jobs for about 100 million family units. Its economic impact is estimated to be approximately $500 billion per year worldwide^1^. Furthermore, cotton is a major economic driver for some developing countries. Besides fibre, cotton is the third largest field crop in terms of edible oilseed tonnage in the world. In addition to its 21% oil content, cottonseed is a source of relative high quality protein (23%). Global cottonseed production can potentially provide the protein requirements for half a billion people per year if the seed is safe for human consumption^2^. Biologically, cotton fibre is an excellent model system for the study of plant cell elongation, cell wall and cellulose biosynthesis^3^. Moreover, the fundamental study of cellulose biosynthesis in fibre cells may lead to the improvement of diverse bioenergy crops^1^.

The most widely cultivated cotton species today are tetraploid *Gossypium hirsutum* and *G*. *barbadense*, both of which originated from inter-genomic hybridization between an A-genome-like ancestral African diploid and a D-genome-like American diploid^4^,^5^. As the donor of the tetraploid, the diploid A genome species produce spinnable fibre, whereas the D genome species do not^6^. However, intensely directional selection of AD tetraploid cotton by humans has consistently led to more superior yield and/or quality characteristics than the A genome diploid cultivars. Selective breeding of *G*. *hirsutum* (AADD, AD1 genome) has emphasized maximum yield, whereas *G*. *barbadense* (AADD, AD2 genome) is prized for its superior length, strength, and fineness of fibre. Although recent whole genome sequencing analyses of diploid cottons (*G*. *raimondii*^7^,^8^ and *G*. *arboreum*^9^) and tetraploid *G*. *hirsutum*^10^,^11^ have provided valuable reference genomes for cotton and a number of shotgun sequencing^12^ efforts have increased our understanding of variations in the cotton genomes, our understanding of the impact of allopolyploidization on fibre production and quality at the genomic level remains minimal. To understand how allopolyploidization has led to desirable agronomic traits, it is necessary to uncover the sequences of the complete tetraploid cotton genome and to identify the specific genes involved in its superior fibre development. Furthermore, tetraploid cotton also represents an interesting biological system to study the interplay between co-residing subgenomes and the evolution of allopolyploid speciation.

Here, we report the genome sequence of the superior fibre quality tetraploid cotton, *G*. *barbadense* acc. 3-79 using a whole-genome shotgun approach with large fragments of DNA Paired-End Tag (DNA-PET) sequencing data. We assembled high quality scaffolds and assigned most of them to the corresponding A-subgenome (At) and D-subgenome (Dt). The final genome assembly was 2.57 Gb, with 80,876 protein-coding genes and ~69.11% repeats of the genome. Using the assembled tetraploid genome, we investigated the potential impacts of allopolyploidization on the dynamics of sub-genomic changes and expression partitions of homoeologous genes, particularly those involved in fibre development. The *G*. *barbadense* genome sequence and our analyses presented in this study provide a new set of valuable resources, which can be of great importance to identify candidate genes and to study their interplay for improving the cotton fibre quality and productivity. We believe that the characterization of the tetraploid *G*. *barbadense* genome not only represents a major advance in our understanding of the dynamics of the cotton genome, but also provides novel insights into the mechanism of polyploidization in plants.

## Results

### Genome assembly and annotation

Using a combined whole-genome shotgun approach and DNA-PET sequencing with insertion sizes ranging from 500 bp to 20 Kb in length, we generated 470.93 Gb of raw sequence reads (Supplementary Table 1). Based on *k*-mer analysis of total short sequencing reads, we estimated that the size of the *G*. *barbadense* genome is approximately 2.57 Gb (Supplementary Figure 1), an estimate which is slightly larger than that previously proposed (2.45 Gb^13^) and is the same as the recently estimated tetraploid *G*. *hirsutum* genome^11^. After filtering, we obtained 446.58 Gb of high-quality sequencing data, representing a 173-fold base-pair coverage and a 5,846-fold physical coverage of the *G*. *barbadense* genome (Supplementary Table 1). Using SOAPdenov^14^, we assembled the sequence reads into 29,751 scaffolds (length from 1 Kb to 2.15 Mb; N50 = 260.06 Kb) covering the whole estimated genome with gap of 334.55 Mb (Table 1). Comparison with the recently published two *G*. *hirsutum* genomes, the assembly length without gap is less than the Zhang *et al.* estimate (2.4 Gb)^11^ and a little longer than that of Li *et al.* (2.17 Gb)^10^.

To assess the accuracy of the assembled scaffolds, 144 public Bacterial Artificial Chromosome (BAC) clone sequences of *G*. *hirsutum* were aligned to the *G*. *barbadense* genome using lastz software in the first instance. The results showed that, there are only 115 BACs which have matched to the assembled scaffolds with a low coverage of 43.9% and a similarity of 96.3% (Supplementary Table 2). These BAC sequences were also aligned to the genomes of *G*. *raimondii* and *G*. *arboreum*. The coverage of *G*. *barbadense* is higher than of the *G*. *arboreum* genome, and lower than for the two *G*. *raimondii* genomes. However, all coverage is less than 70.0%. Taking account of the fact that *G*. *hirsutum* and *G*. *barbadense* are two different tetraploid cotton species, we constructed a BAC library of *G*. *barbadense* and sequenced 10 randomly chosen BAC clones. Alignment these BAC sequences against scaffolds of *G*. *barbadense* showed that nine of ten BAC sequences were matched to single scaffolds with more than 90.5% coverage and 99.5% identity on average, except for BAC06 (Supplementary Figure 3; Supplementary Table 3). We also used the same method to do the comparison between BAC sequences and scaffolds from *G*. *raimondii* or *G*. *arboreum*. The average coverage of *G*. *arboreum* and *G*. *raimondii* are 84.6% and 92.7% respectively, and the average identities are 98.2% and 99.5% respectively. To further evaluate the assembly quality, 17,894 expressed sequence tags (ESTs) and 1,959,060 454/Roche transcriptome sequences were mapped to the genome scaffolds. Sequence alignment results showed that 93.9% of ESTs and 96.7% of the 454 sequences were covered by the assembly (Supplementary Table 4). These results suggest that the assemblies has extensive genome coverage with high accuracy.

**Figure 1:**
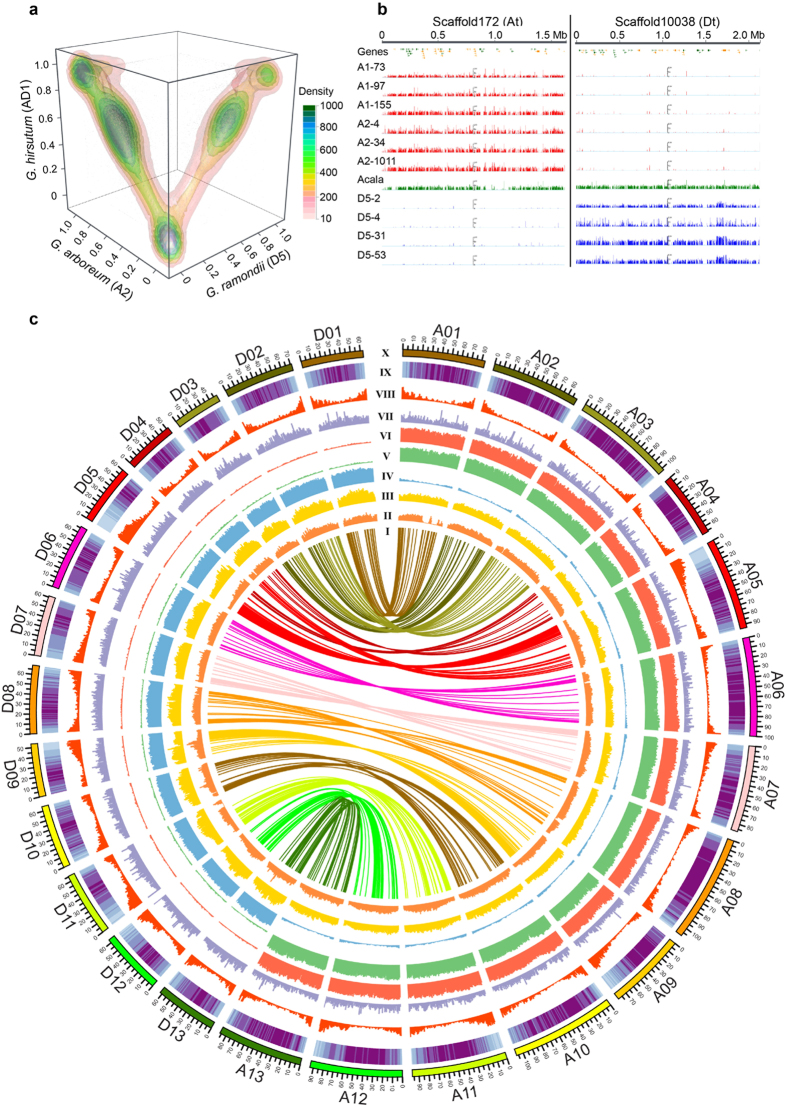
Assembling the allotetraploid genome of *G. barbadense*. (**a**) Three-dimensional scatter plot with density contour shows the mapping base pair coverage of shotgun sequencing reads derived from *G. arboreum*, *G. raimondii* and *G. hirsutum* to the assembled scaffolds of *G. barbadense* tetraploid genome. (**b**) Examples of *G. barbadense* scaffolds assigned to At or Dt by mapping of shotgun sequencing reads from diploid progenitor genomes (red tracks for A genome clade; green track for tetraploid AD1 genome; blue tracks for D5 genome clade). Mapping density scale is the same (maxima 100 in each track) for all tracks. (**c**) Circos plot shows the genome-wide alignments between the TM-1 genome^11^ and the two subgenomes of *G. barbadense*. I: syntenic alignments between the At, Dt and TM-1 genome; II: SNP density between *G. barbadense* and *G. hirsutum* (window size is 1 Mb); III: SNP density in At (*G. barbadense* vs. *G. arboreum*) and in Dt (*G. barbadense* vs. *G. raimondii*). IV-VII: coverage of *G. raimondii*, *G. herbaceum*, *G. arboreum* and *G. hirsutum* mapped to the scaffolds of *G. barbadense*, respectively; VIII: gene density; IX: TE density; X: the length of pseudo-chromosome.

In order to assign assembled scaffolds to a particular subgenome, we mapped 11 public genome shotgun sequencing datasets^12^ to *G*. *barbadense* scaffolds (Supplementary Table 5), including the A1, A2, D5 and the tetraploid AD1 genomes. When we mapped shotgun sequencing reads of A2 and D5 genomes to all the assembled *G*. *barbadense* scaffolds, we noted that indeed the scaffolds were separated into two distinctive groups through the mapping coverage (Supplementary Figure 4b). The mapping of *G*. *barbadense* scaffolds by the shotgun reads of another tetraploid *G. hirsutum* (AD1 genome) yielded two corresponding groups of scaffolds, one associated with the A2 genome and the other one with the D5 genome, respectively. As expected, the AD1 genome sequence reads were mapped to both of At and Dt specific scaffolds (Fig. 1a). In our control procedures, the mapping of the A1 and A2 genome sequences were highly correlated with each other (Supplementary Figure 4a), primarily due to the close relationship between the A1 and A2 genomes^12^. Based on the mapping and base-pair coverage analyses of A1/A2 and D5 genome shotgun sequence reads (Supplementary Figure 4c), we assigned 14,319 scaffolds to At and 6,967 scaffolds to Dt (Supplementary Table 6). The remaining scaffolds (8,465) that failed to meet our criteria were marked as “ungrouped”.

Figure 1b showed the example of how to assign the scaffold to At and Dt, with sequences mapped by the different cotton genome sequence reads. All the mapping details of 29,751 scaffolds were shown by the Generic Genome Browser (GBrowse) of the genome website (http://cotton.cropdb.org/). Most of the “assigned” scaffolds are longer (~100 Kb) than the “ungrouped” scaffolds (Supplementary Figure 4d). The total length of the scaffolds assigned to At is 1.50 Gb and the total length of those assigned to Dt is 853 Mb. In contrast, the ungrouped scaffolds represented only 226.7 Mb (8.8% of total scaffold length) and most of them contained a high number of transposable elements (TEs) and few genes (Supplementary Figure 4e).

Based on the subgenome assignment, a recently published ultra-high density genetic map^15^ was used to construct the *G*. *barbadense* pseudochromosomes. We were able to anchor 7,841 scaffolds with a total length of 1,195 Mb in the appropriate order to At (representing 80.0% of the At and including 32,530 genes) and 4,476 scaffolds with a total length of 802 Mb to Dt (representing 94.0% of the Dt and including 32,321 genes) (Fig. 1c; Supplementary Table 6 and Supplementary Table 7). The remaining unanchored scaffolds were typically comprised of a high percentage of TEs and a small number of genes (Supplementary Figure 4e), rendering it difficult to anchor these scaffolds to the reference chromosomes.

Using combined gene prediction programmes and transcriptome sequences, we identified 80,876 protein-coding genes in tetraploid cotton, including 36,947 genes in At and 34,575 genes in Dt (Table 1). The number of protein-coding genes is a little larger than suggested by Li *et al.* (70,478)^10^ and Zhang *et al.* (76,943)^11^ for *G. hirsutum*. The gene transcripts have an average length of 3,223.6 bp with a mean coding sequence (CDS) size of 1,164.1 bp and an average of 5.2 exons per gene (Supplementary Table 8). Over 91.8% of the predicted coding sequences were supported by RNA-Seq data (Supplementary Table 9). Among the annotated genes, approximately 95.6% of the predicted genes have homologue matches in public databases: 90.1% proteins show homology to proteins in the TrEMBL database, 76.9% are identified in InterPro, and 60.5% are assigned at least one Gene Ontology (GO) terms (Supplementary Table 10).

### Genome expansion of At subgenome

Transposable elements (TEs) are an important genomic feature in plant genomes and play important roles in driving genome evolution. In total, we identified 1,778.6 Mb of TE in *G*. *barbadense* (69.1% of the tetraploid cotton), including 1,098.0 Mb of TE sequences in At (representing 73.5% of the subgenome) and 541.6 Mb of TE sequences in Dt (representing 63.5% of the subgenome) (Table 1). In the recently published *G*. *hirsutum* genome, 1,339 Mb (64.8%)^11^ and 1,445 Mb (66%)^10^ were identified respectively. The difference was most likely caused by using the different databases. In this study, the public database Repbase^16^ and *de novo* TE seed libraries were also used. The database of the Munich Information Center of Protein Sequences (MIPS) was only used to identify the TE sequence in the Zhang *et al.*^11^. In another study, the Repbase and *ab initio* databases were used for identifying TEs^10^, however different and less extensive software was used for identifying TE seed sequences.

**Figure 2:**
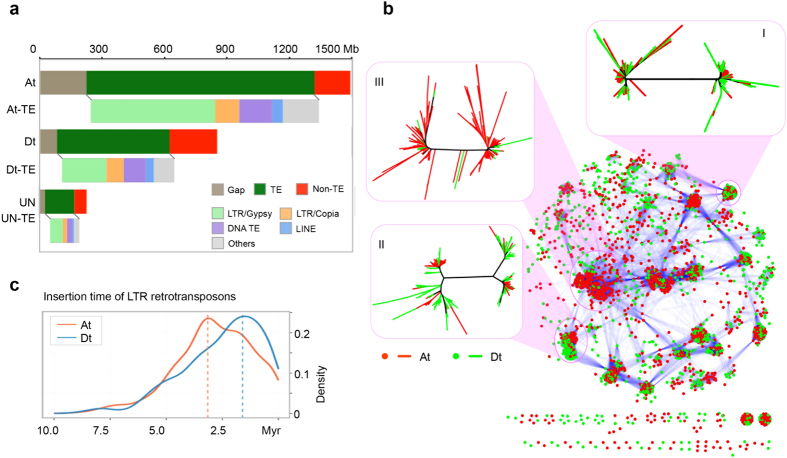
Expansions of LTR retrotransposons in the At subgenome of *G*. *barbadense*. (**a**) Distribution of different classes of transposable elements. (**b**) LTR retrotransposons are clustered into putative families according to reciprocal blast similarity. The *Gossypium* lineage shared (I), divergent (II) and At lineage specific (III) LTR transposons are enlarged. (**c**) The insertion time of LTR retrotransposons in At and Dt respectively.

We have further analysed Long Terminal Repeat (LTR) retrotransposons (Supplementary Table 11), observing that the *Gypsy* superfamily of LTR retrotransposons accounted for 599.6 Mb (40.2%) of At, which is substantially higher than that observed in Dt (214.2 Mb, 25.1%) (Fig. 2a; Supplementary Table 11) in common with *G. raimondii*^7^,^8^, *G. arboreum*^9^ and *G. hirsutum*^10^,^11^. The clustering patterns indicate that specific LTR retrotransposon clusters preferentially occurred in At, with the exception of several shared clusters (Fig. 2b). The cluster of *Gypsy* enriched in At was classified into the subclades of Peabody and Retrosat2 in the Del clade, and Athila in the Athila/Tat clade (Supplementary Table 12). It is documented that the Gypsy of G45 and G84 is specific in to the *G*. *barbadense* genome^17^, and Gorge3 expansion is typical in large genome size cotton species^18^. The phylogenetic tree showed that G45 is most closely related to Peabody, although very close to Retrosat2 (Supplementary Figure 5). Gorge3 of At has 96.1% and 80.9% amino acid sequence identity with G45 and G84, respectively^18^. We deduced that G45 and Gorge3 are also part of the Del clade.

The timing of insertion for LTR retrotransposons points to a very recent burst of LTR retrotransposon amplification in Dt, peaking within 1.9 million years (Myr) ago, whereas, At appeared to have undergone a surge of retrotransposon amplification approximately 3.1 Myr ago (Fig. 2c). The results suggest that most expansions of extant LTR retrotransposons independently occurred after the lineage separation but before allotetraploidization. We observed higher ratios of solo and truncated LTRs to intact LTR in At than Dt (Supplementary Table 13), while Dt and its diploid progenitor have similar ratios of solo and truncated LTRs to intact LTR in their genome/subgenome (Supplementary Table 13).

### Dynamic variation of genome architecture impacted by allopolyploidization

Allopolyploidization, which results in whole chromosome doubling through the hybridization of related species, would have a profound impact on the architecture of the genome^19^. Some of these changes have been linked to novel phenotypic variations in polyploids^20^. Relative to diploid progenitor cottons, tetraploid cottons (*G. hirsutum* and *G. barbadense*) have developed superior fibre phenotypes, representing the development of new agronomic traits. Assembling the genome of the tetraploid cotton *G. barbadense* provides a new avenue to investigate the dynamics of the genome during cotton allopolyploidization.

**Figure 3:**
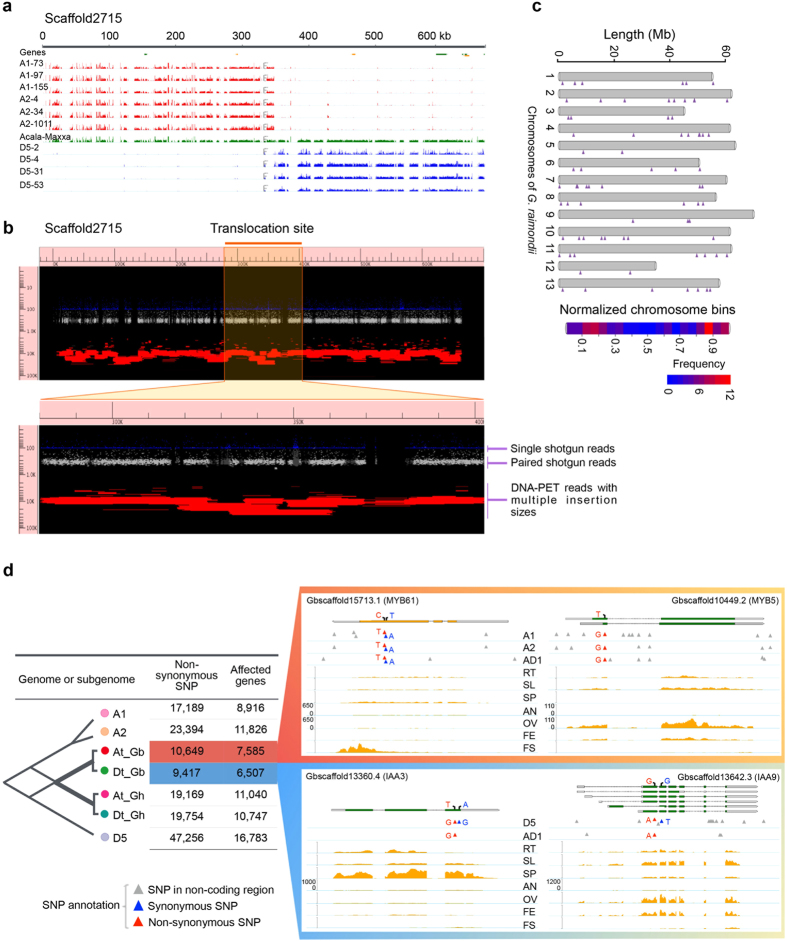
Impact of allotetraploidization on genomic variation. (**a**) An example of At-Dt-hybrid scaffold. The mapping tracks, order and density scale are same as in Fig. 1b. (**b**) Evaluating assembly quality of hybrid scaffold at junction region. The y-axis indicates that the fragment size of paired-end reads in log scale, the x-axis indicates the length of the scaffold. As shown, single-end shotgun reads (blue, ~100 bp) and paired-end shotgun reads (white, ~300 bp) were clustered into contigs, which were further connected by abundant DNA-PET reads (size range of 5, 10, and 20 Kb) in this scaffold. (**c**) Genome-wide distribution of inter-subgenome translocations. Using the D5 genome reference as a framework, all 77 putative translocation sites are indicated along the 13 chromosomes by triangles. (**d**) Lineage-specific SNP divergence in diploid genomes and tetraploid subgenomes. The right panel provides examples of genes that were affected by lineage-specific non-synonymous SNPs in subgenomes of *G. barbadense* (two *MYB* genes holding lineage-specific SNPs in At and two auxin-responsive factor genes holding lineage-specific SNPs in Dt). The RNA-Seq tracks show the expression profile of the four genes (the details of sample was listed in the Supplementary Table 10) and adjusted on the same scale.

Intriguingly, we identified 77 that contained hybrid sequences in the “ungrouped” scaffolds, i.e. one part from At and the other part from Dt (Fig. 3a). Such At-Dt-hybrid scaffolds could have emerged as a result of inter-subgenome translocation events occurring in tetraploid genome post-polyploidization. Considering that the hybrid scaffolds might also be caused by artifacts (such as assembly errors), we manually evaluated the assembly of these scaffolds and verified that there were abundant paired-end reads with different lengths covering the At-Dt junction regions (Fig. 3b). The sequencing of PCR products also validated the hybrid scaffolds (Supplementary Table 20). Using the D5 genome as a reference, we demarcated the putative translocation sites to the 13 pseudochromosomes of Dt and observed that chromosomal translocations occurred more frequently at sub-telomeric regions (Fig. 3c). These results, combined with previous observations^21^ and recently published *G*. *hirsutum* genome^10^, suggest that inter-genomic concerted modification is an important dimension of polyploidy evolution.

In order to study genomic divergence, we mapped genome shotgun sequence data from the diploid progenitors (A1, A2, and D5) and the tetraploid *G*. *hirsutum* (AD1) to At and Dt of *G. barbadense* to identify SNPs. As expected, the genomic divergence between the two tetraploids (*G. barbadense* and *G. hirsutum*) was less than the divergence between the tetraploid and its diploid progenitors. Interestingly, we found that the divergence of Dt from its proposed progenitor D5 was significantly larger than that of At from its proposed progenitor A1 or A2 based on the SNP density (Supplementary Table 14). This may suggest that Dt in tetraploid *G. barbadense* could have a higher mutation rate or that At could be under the constraints of higher selective pressure.

To survey the genome-wide landscape of genomic variation, we obtained the SNP distribution density for each of the 26 assembled pseudochromosomes (Fig. 1c). Most of the SNPs in At and Dt of *G. barbadense* were evenly distributed against their progenitors A2 and D5. However, the distributions of SNPs against the *G. hirsutum* tetraploid AD1 genome were notably uneven. The most remarkable low-density regions were located in A01, A06, A08, D04 and D11 (Fig. 1c). A recent study also detected the SNP-poor regions on the corresponding region of chromosomes A01, A05, A06, A08, D04, and D11^15^, but the region of A05 was not detected in this study. These regions of reduced SNP diversity could represent possible selective sweeps due to common positive selection pressure exerted on both tetraploid cottons that may have occurred after allotetraploidization. Considering the length of time over which both tetraploid cottons have been cultivated, these conserved regions may also be landmarks of domestication or the historical introgression between *G*. *hirsutum* and *G*. *barbadense*. There are also 4 high-density regions, which are all located in Dt, such as D09, D10 and D13. These regions may indicate the evolutionary divergence between *G. barbadense* and *G. hirsutum*, and contribute to phenotypic differences.

In order to assess specific genetic variations that may have contributed to the novel phenotypes that only arose in tetraploid cottons, it is necessary to analyse lineage-specific genetic variations between the tetraploid subgenomes and their diploid progenitor genomes. We conducted an all-vs-all comparative analysis and identified lineage-specific SNPs, including 1.5 million At-specific SNPs and 0.98 million Dt-specific SNPs (Supplementary Table 15 and Supplementary Table 16). To further explore the functional effects of lineage-specific variations, we focused our analysis on identifying lineage-specific non-synonymous SNPs and the affected protein-coding genes (Fig. 3d). In *G. barbadense*, we identified 7,585 and 6,507 protein-coding genes that carry lineage specific non-synonymous variations in At and Dt respectively. GO enrichment analysis showed that genes in Dt affected by lineage-specific non-synonymous SNPs were significantly (*p*-value = 0.042) enriched for a GO functional item representing response to hormonal stimulus, consistent with recent reports that AUX-IAA target genes are involved in controlling multiple pathways in developing cotton fibre cells^22^. Recent studies have suggested that *MYB* genes play an important role in cotton fibre development^23^,^24^,^25^,^26^. In At, we found that, out of a total of 131 MYB transcription factors (TFs), 31 genes of the MYB family were affected by lineage-specific SNPs. In addition, nearly half of these affected *MYB* genes were specifically expressed at different stages of fibre development (Supplementary Figure 8d). The phylogenic tree of the promoter region of the 31 *MYB* genes shows that the distributions of MYBs which are dominantly expressed in fibre tissue were not clustered together (Supplementary Figure 9). These results suggest that lineage-specific divergences may have specific physiological or functional contributions to the novel phenotypic variations in allotetraploid cottons.

### Dynamic changes of gene content

In addition to impacting the architecture of the genome, allopolyploidization could also have “genome shock” effects resulting in potentially dramatic evolutionary adaptations, including gene expansion/contraction and could also contribute to the evolution of gene expression and sub-functionalization. Using the OrthoMCL programme^27^, we identified 20,378 orthologous gene clusters in the tetraploid At, Dt and the diploid D5 genome, and classified them into nine groups based on gene family expansion/contraction patterns for At and Dt in reference to the D5 genome (Supplementary Figure 7a).

More than half of the orthologous gene clusters (10,695) displayed no differences in the number of genes among the three genomes, suggesting that allopolyploidization had no effect on these genes. Among the other classes, the largest numbers of genes were observed in gene families that showed contraction of gene family members relative to the D5 genome (6,502 orthologous gene clusters), some of which showed contraction in At- and Dt-specific genes. For others, contraction was limited to either the At or Dt genes. These observed patterns may be expected due to gene redundancy in the allopolyploid. Interestingly, we also identified a group of orthologous gene families that are expanded in the allopolyploid cotton genome, either only in At (1,083 orthologous gene clusters) or in Dt (1,122 orthologous gene clusters), or both (359 orthologous gene clusters). We noted another small group of orthologous gene families that was expanded in one subgenome yet contracted in the other subgenome. These data suggest that a substantial number of orthologous genes were affected by the impact of allopolyploidization.

**Figure 4:**
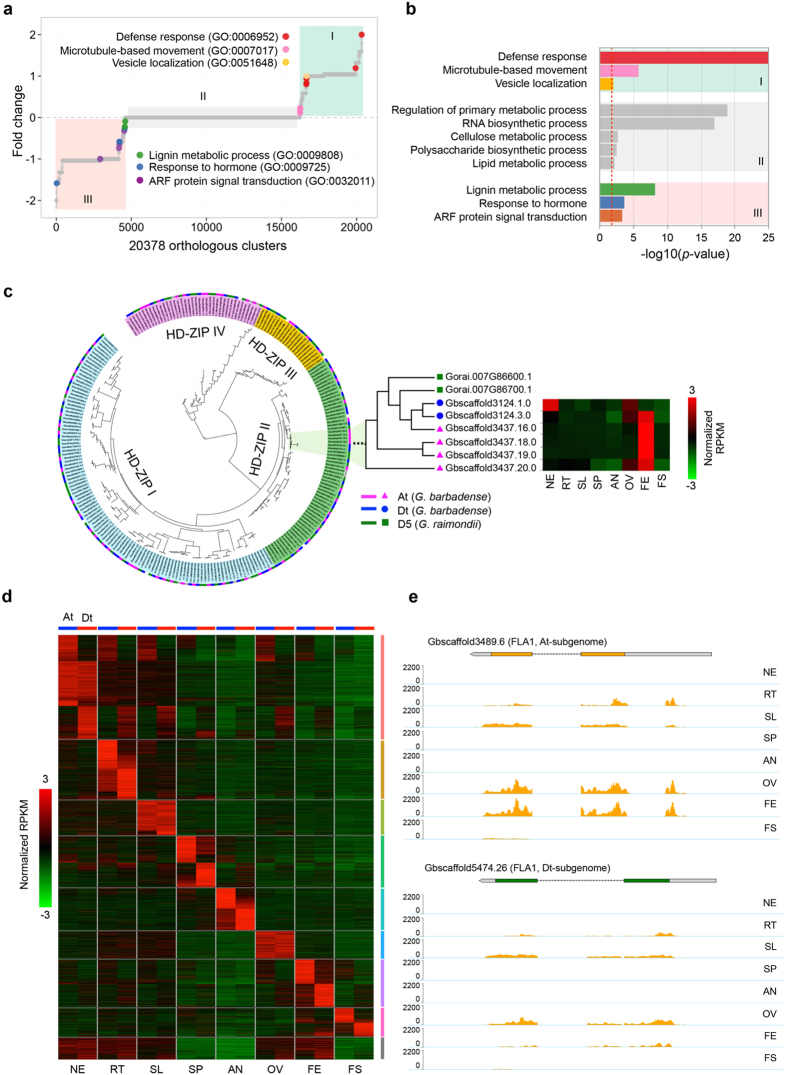
Dynamic changes of genes and their expression in allotetraploid subgenomes of *G. barbadense*. (**a**) Dot plot shows the number of orthologous genes in subgenomes. The y-axis represents the log_2_ ratio of gene number changes of At versus Dt using the D5 reference genome as normalizer. The x-axis represents the number of orthologous gene clusters. Expanded orthologous gene clusters with biologically relevant functions (GO terms) are indicated using colored dots. (**b**) Enriched representatives of GO items of genes expanded in At (category I), expanded in Dt (III), and unchanged (II) orthologous gene clusters. The dotted red line indicates the GO enrichment cutoff at *p*-value = 0.01. (**c**) Phylogenetic tree of HD-Zip genes from *G. barbadense*, *G. raimondii*, and *Arabidopsis*. Highlighted is a sub-phylogenetic clade. Right panel is the expression profile of corresponding HD-Zip genes. (**d**) Transcriptional divergence of homoeologous gene pairs in *G. barbadense*. Heat map shows normalized expression levels for 6,461 paired homoeologous genes. (**e**) An example of homoeologous gene pairs and their differential expression patterns.

We were particularly interested in those genes that were expanded in tetraploid post-allopolyploidization, since their functions could be more directly related to the novel phenotypes that arose in tetraploid cottons (Fig. 4a). Using GO enrichment analysis, we observed that expanded genes in At were enriched in items related to defense response, microtubule-based movement and vesicle localization, while the expanded genes in Dt were enriched for items related to lignin metabolic process, response to hormone and ARF protein signal transduction (Fig. 4a,b). Based on the analysis of lineage-specific variations and orthologous gene family expansions, we found that genes involved in responses to hormones were dramatically altered in Dt, suggesting that Dt may play a specific physiological role in *G. barbadense*. A recent study showed that the D genome provides many fibre genes after its unification with the parental diploid cotton A genome, although the D genome itself does not produce any spinnable fibre^28^.

Local gene duplication is one of the mechanisms for gene family expansion^29^. The HD-Zip transcription factor (TF) family is unique to the plant kingdom^30^ and recent studies have demonstrated that it plays a pivotal role in cotton fibre development^31^,^32^. We identified a total of 149 HD-Zip genes in *G. barbadense*, 65 of which were assigned to At and 73 to Dt, while the remaining 11 HD-Zips were ambiguous and could not be assigned to either subgenome. Overall, the number of HD-Zip genes in each of the two subgenomes in the tetraploid is roughly the same as the number of HD-Zip genes (79) in the D5 diploid genome (*G. raimondii*). However, at the subclade level, we observed an unbalanced gene amplification event in At of *G*. *barbadense*. As highlighted in Fig. 4c, a subclade includes two HD-Zip genes in the diploid D5 genome and two in the Dt of *G*. *barbadense*. However, we observed that four HD-Zip genes were included in At, suggesting that an amplification of the HD-Zip genes had occurred in At but not in Dt of *G*. *barbadense*.

Micro-synteny blocks showed that these four copies of the HD-Zip genes in At of *G*. *barbadense* were organized in tandem at a close distance, while there are only two copies of HD-Zip genes in Dt of *G*. *barbadense* and in the D5 genome in the corresponding synteny block (Supplementary Figure 6). These observations suggest that the two HD-Zip genes in Dt were inherited from its progenitor D5 genome and that the four HD-Zip genes in At involved a gene amplification event by tandem duplication. It is likely that this tandem duplication event occurred after the divergence of A and D genomes from their common ancestor. It is also possible that this duplication took place post-allotetraploidization.

To further understand the potential functions of these HD-Zip genes, we analysed their expression patterns using RNA-Seq data generated from different tissues of *G. barbadense*. Interestingly, while the four HD-Zip genes in At are all expressed in the fibre elongation stage in this block (Fig. 4c, right; Supplementary Figure 6b), the two HD-Zip genes in Dt of *G*. *barbadense* were expressed in two different tissues, one in callus and the other one in fibre elongation stage (Fig. 4c, right; Supplementary Figure 6c). This pattern of gene expression suggests that the HD-Zip genes in Dt exhibit a functional divergence (neofunctionalization) although it is not clear when this occurred in the evolution of the D genome lineage. In contrast, the four duplicated HD-Zip genes in At had enhanced HD-Zip functions during the fibre elongation stage (FE) (Fig. 4c), possibly resulting from their recent recruitment for the important biological function that accompanied by allotetraploid formation and evolution in *G. barbadense*. Other examples of gene families that were potentially involved in fibre development are shown in Supplementary Figure 10.

To further investigate potential effects of allopolyploidization on homoeologous gene functions in the tetraploid genome, we identified 6,461 pairs of homoeologous genes by analyzing collinear synteny blocks between the At and Dt. We surveyed their gene expression profile in eight different tissues and stages of fiber development using RNA-Seq data and k-means clustering analysis. Surprisingly, we observed that many of the highly expressed homoeologous gene pairs showed biased tissue-specific patterns (Fig. 4d,e; Supplementary Table 17). We identified 708 and 425 homoeologous gene pairs that were highly and specifically expressed in the fiber elongation (FE) and secondary cell wall synthesis (FS) stages, respectively. Out of these, more than half (58% for FE and 67% for FS) were exclusively either At-biased or Dt-biased. This phenomenon may suggest extensive collaboration between the co-residential sub-genomes in a complimentary manner, which could provide a mechanistic basis for the functional advance that led to novel and superior agronomic traits in allopolyploid cotton.

### CesA “relay race” model for fibre development in allotetraploid cotton

**Figure 5:**
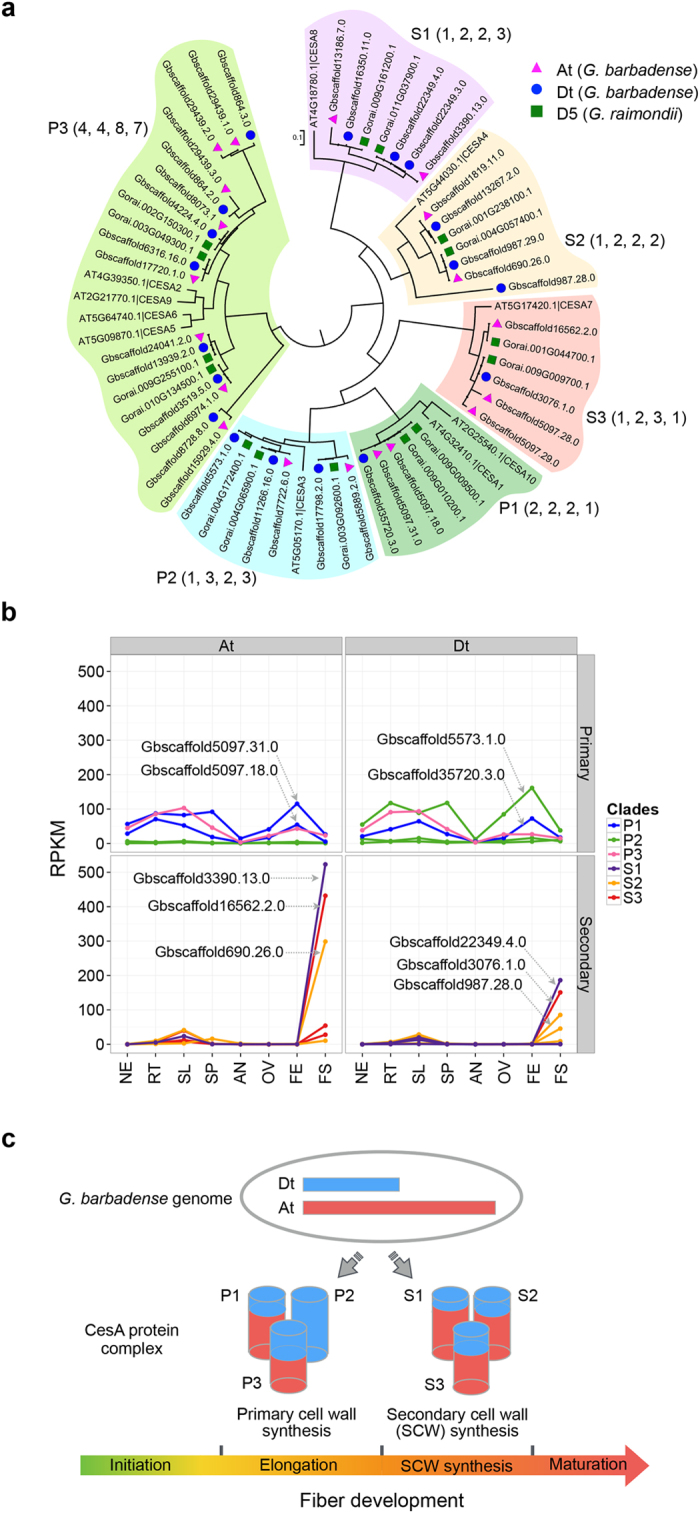
Subgenomic contribution of *CesA* functions to fibre development in allotetraploid *G. barbadense*. (**a**) Phylogenetic tree of *CesA* orthologous gene family from *G. barbadense*, *G. raimondii*, as well as *Arabidopsis*. According to their function in *Arabidopsis*, *CesA*s were grouped into primary (P1 to P3) and secondary (S1 to S3) clusters in cell wall cellulose biosynthesis. In each of the clusters, the numbers of *CesA* genes are shown in parentheses in the order of *Arabidopsis*, D5 genome, At and Dt. (**b**) Expression profiles of *CesA* genes in both At and Dt of *G. barbadense*. The expression profiles of major contributing genes are indicated with their gene IDs. For the P3 group, the expression data of eight *CesA* genes in At (up-left) and the 7 *CesA* genes in Dt (up-right) are summed to show expression profile, respectively. (**c**) A proposed CesA “relay race” working model for fibre development in allotetraploid cotton.

It is known that the *CesA* gene family encodes a complex of cellulose synthase regulating the biosynthesis of cellulose that is dominant in cotton mature fibre. There are 10 *CesA* genes in *Arabidopsis*. At least three *CesA* genes from the group including *CesA1*, *CesA3*, *CesA2* or *CesA6* are required for primary cell wall synthesis^33^. *CesA1* and *CesA3* appear to be absolutely required, whereas *CesA2* and *CesA6* may be at least partially redundant. Three distinct *CesA* genes (*CesA4*, *CesA7* and *CesA8*) are required for the cellulose synthesis of secondary cell wall^34^. In *G. barbadense*, we identified a total of 37 *CesA* genes, 19 of which were assigned to At and 18 of which were assigned to Dt. The number of *CesA* genes in each of the two subgenomes was comparable to the 15 *CesA* genes found in the D5 genome. Our phylogenetic analysis indicated that the 37 *G. barbadense CesA* genes were grouped into six clades, which included the corresponding orthologs from *G. raimondii* and *Arabidopsis* (Fig. 5a).

Analysing the number of *CesA* genes representing different genomes and distributed in each of the phylogenetic and functional groups yielded some interesting observations. In all these clades except for P1 and P3, *G. raimondii* genome had more *CesA* genes than did *Arabidopsis*. The tetraploid At and Dt had similar numbers of *CesA* genes as the D5 genome with minimal (±1) deviation. However, the P3 group included four *CesA* genes from *Arabidopsis*, four from D5 genome of *G. raimondii*, eight from At and seven from Dt of *G. barbadense*. The P3 *CesA* genes in both At and Dt were surprisingly and convincingly doubled in tetraploid cotton, suggesting that possible duplication events took place post-allopolyploidization. We also investigated the *CesA* gene family of *G*. *arboreum* and the two recently published *G*. *hirsutum*. There are 6 genes of P3 *CesA* in the *G*. *arboreum* genome. The gene numbers of At and Dt described by Li *et al.* are 6 and 5^10^, and by Zhang *et al.* are 5 and 6 respectively^11^ (Supplementary Table 21). The gene number of P3 in diploid A genome, At and Dt of *G*. *barbadense* are all more than for the D genome.

In order to decipher comprehensively the complex role of *CesAs* for cotton fibre development, we analysed the expression profiles of the 37 *CesA* genes in *G*. *barbadense* using RNA-Seq data in eight tissues (Supplementary Table 9). First, we found that all *CesA* genes involved in primary cell wall synthesis (P1, P2, and P3) are expressed at the fibre elongation (FE) stage and at the fibre secondary (FS) cell wall synthesis stage (Fig. 5b; Supplementary Table 18). For those in At, the two P1 *CesA* genes were the most active, while the two P2 *CesA* genes were demonstrated to have only basal levels of expression. In contrast, in Dt, one of the P2 *CesA* genes was predominantly expressed specifically during FE whereas expression of the other P2 *CesA* remained at basal levels, as did its homologous counterpart in At of *G*. *barbadense*.

We also noted that, individually, the eight P3 *CesA* genes in At and the seven P3 *CesA* genes in Dt of *G*. *barbadense* did not display high levels of expression (Supplementary Table 18). Collectively, however, the sum of their expression was comparable to expression levels of the P1 *CesA* genes in At and Dt of *G*. *barbadense* (Fig. 5b). Second, we found that all of the *CesA* genes involved in secondary cell wall synthesis in three functional groups (S1, S2, and S3) were almost exclusively and highly expressed during FS and the At specific genes were expressed the most, consistent with previous evidence in *Arabidopsis*^32^. The expression patterns of key *CesA* genes (marked in Fig. 5b) in both subgenomes were verified by quantitative real-time PCR (qRT-PCR) experiments (Supplementary Figure 11; Supplementary Table 19). Intriguingly, it appears that there is a definitive on/off switch for expression between the primary and secondary *CesA* genes that is triggered during the transition from cotton fibre elongation to secondary cell wall synthesis.

Given these findings, we propose a “relay race” model for fibre development involving the *CesA* genes in the tetraploid cotton *G. barbadense* (Fig. 5c). In this working model, the P1, P2 and P3 *CesA* transcripts conduct primary cell wall biosynthesis by working together as a complex team in which the At-specific *CesA* genes may contribute 2/3 of the P1 transcripts and the Dt-specific *CesA* gene may provide 1/3 of the P1 transcripts. The Dt-specific *CesA* gene contributes all P2 transcripts, whereas *CesA* genes in both At and Dt contribute P3 transcripts. During FE, the primary cellulose machinery reaches its peak activity, most notably for P2 *CesA* gene activity in Dt of *G*. *barbadense*. At the transition from fibre elongation to secondary cell wall synthesis, fibre cellulose biosynthesis is rapidly overtaken by the machinery of secondary cell wall synthesis comprising of protein complexes that include S1, S2 and S3, most of which are contributed by At-specific *CesA* genes with the *CesA* genes residing in Dt also providing meaningful portions.

## Discussion

Modern DNA sequencing technology and computational programs for genome assembly have advanced our ability to generate a large volume of high-quality, short-sequencing reads and to assemble them into long contigs and scaffolds for complex genomes, including those of many plants. However, the complexity of allopolyploid crops has represented a challenge for the genomics community due to the co-residence of closely related homoeologous subgenomes; thus the study of many important crops including cotton has been hindered. Ideally, well-established high density genetic linkage maps would be very valuable in assembling these genome sequences at the level of the chromosome. The allotetraploid cotton *G. barbadense* is derived from the interspecies hybridization of extant A-genome and D-genome progenitors. The D5 genome (*G. raimondii*) and A2 genome (*G. arboreum*) had been recently sequenced and assembled as the reference genome for cotton^7^,^8^,^9^, and shotgun sequencing efforts have generated about 20X coverage reads for several lines of A1 (*G. herbaceum*) and A2 (*G. arboretum*) genomes^12^. In this study, we first used the progenitor of A-genome and D-genome sequence reads to assign the assembled *G. barbadense* scaffolds on the basis of their origin to either the At or Dt. This approach was effective as evidenced that the total length of the assigned scaffolds corresponded well to the length of the designated subgenomes (1.5 Gb of scaffolds were assigned to the At and 853 Mb were assigned to the Dt). Then, we constructed 26 pseudochromosomes (At, 1,195 Mb; Dt, 802 Mb) using the recently released high-dense genetic and physical maps^15^. We believe that the strategy we describe here is applicable to other polyploidy plant genomes.

Our analysis of genomic variation revealed that the architecture of the tetraploid cotton genome has gone through extensive changes through inter-subgenome translocation and nucleotide variation post allopolyploidization, possibly leaving the marks of domestication on the genome of tetraploid cottons. Such variations could have profoundly impacted gene function and could have contributed to the emergence of novel phenotypes. Through our analyses of lineage-specific non-synonymous variations, we have identified thousands of genes in both of the At and Dt that could have undergone functional changes. Based on orthologous relationships, we were able to measure the “genome shock” effect on gene family expansion and contraction in allopolyploidization. More importantly, we have observed that many homoeologous gene pairs in the At and Dt have distinctively biased expression patterns in different tissues and in different stages of fiber development. This observation suggests that substantial sub-functionalization of homoeologous genes occurred post allopolyploidization in tetraploid cotton. These results led us to identify possible functions for each member of the *CesA* gene family and enabled us to postulate a collaborative and temporal working model, “relay race”, to explain the mechanism of cellulose biosynthesis in cotton.

This study represents the first step in the direct analysis and serious characterization of the tetraploid cotton genome. The further analysis of these genomic data and the additional characterization of the homoeologous genes and their functions described in this study will provide invaluable insights into the biology and evolution of allopolyploidization in cotton and other plants.

## Methods

### Plant material and genomic DNA preparation

*G*. *barbadense* acc. 3–79 (a doubled-haploid from Pima germplasm) was obtained from the Cotton Research Institute (CRI) of the Chinese Academy of Agricultural Sciences (CAAS) in 2002 and self-fertilized to maintain homozygosity in each generation. Fresh young leaves were collected, immediately frozen in liquid nitrogen and stored at −80 °C until DNA extraction. Genomic DNA was extracted using the standard CTAB method^35^.

### Genome sequencing and assembly

The paired-end (insert size of ~500 bp) and mate-pair (insert size of 5, 10 and 20 kb) libraries were constructed following the standard Illumina protocols. Whole-genome shotgun sequencing was performed with the Illumina Genome Analyzer II System. Raw Illumina reads were first processed by removing low-quality reads, adaptor sequences and possible contaminated reads of bacterial and viral origin. Then, the software QUAKE^36^ was used to correct the reads from each library. The corrected reads were then assembled using SOAPdenovo (-K 63 -R -d), with the three mate-pair libraries being used to link contigs into scaffolds. Scaffolds and contigs were refined further with GapCloser in SOAPdenovo^37^. The flowchart of genome assembly was shown at Supplementary Figure 2.

### Subgenome assignment and pseudochromosome construction

The resequencing datasets of diploid and tetraploid cotton were downloaded from the NCBI SRA database^12^. The shotgun reads of each dataset were mapped to the *G. barbadense* genome assembly using the BWA program with default settings^38^. After mapping, the shotgun reads with low mapping scores (<20) were filtered using SAMtools program^39^. In addition, the redundant reads potentially from PCR amplification were removed using the Picard programme^40^. For each assembled scaffold, the percentages of the base-pairs covered by the shotgun reads from each dataset were calculated using BEDtools^41^.

The following criteria were used to call if a *G. barbadense* scaffold belongs to either At or Dt: 1) more than 40% of a scaffold was covered by shotgun reads derived from diploid A1, A2 or D5 progenitor genomes; 2) the log_2_ ratio of average base-pair coverage between A1 or A2 to D5 diploid progenitor genome is more than 2; 3) the ratio difference is significant (*p*-value < 0.01) as determined by Student’s t-test using the shotgun read datasets derived from multiple lines of each diploid genome (A1, A2, D5) as replicates. Scaffolds that did not satisfy with these three criteria were regarded as “ungrouped”.

A recently published ultra-dense inter-specific genetic map^15^ was used to construct 26 pseudochromosomes. A dataset of SNP segment was truncated from the scaffold according the SNP position information. Each segment has the length of 201 bp, 100 bp from the left of SNP loci and another 100 bp from the right. SNP segments were mapped to the assigned subgenome and ungrouped scaffolds of *G*. *barbadense* using BWA software^38^. The pseudochromosomes were constructed using the following criteria: (1) the SNP segments were unique aligned to the scaffolds with the minimum match of 196 bp; (2) one scaffold was mapped by at least 5 continuous SNP segments; (3) one scaffold was only anchored on one chromosome or one scaffold of TM-1 genome^11^.

### Identification of repetitive elements

Six *ab initio* software packages, LTR_STRUC^42^, LTRharvest^43^, MGEScanLTR^44^, TransposonPSI, RepeatModeler and MITE_Hunter^45^, were used to search for repeat sequences within the *G. barbadense* genome. After removing redundancy with CDHIT^46^, a *de novo* transposable element (TE) library for *G. barbadense* was obtained. We then used RepeatMasker to identify repeats using both the *de novo* library and Repbase^16^. In addition, RepeatProteinMask was used to search the protein database in Repbase against the genome. Identified repeats were classified using software of TEclass^47^ and Repclass^48^.

### TE evolution analysis in *G*. *barbadense*

The LTR_STRUC^42^ program was used to identify full length LTR retrotransposons. LTRs were then classified by aligning the HMM profiles to the GyDB2 database^49^ and according to the rules described by Wicker^50^. We aligned the 5- and 3-ends of the LTR sequences of each retrotransposon using MUSCLE^51^ and calculated the divergence (K) under the Kimura two parameter (K2P) model using the distmat program of EMBOSS toolkit^52^. The divergence time of LTR was estimated using the formula T = K/2r, where r represents a synonymous substitution rate of 1.3 × 10^−8^ per site per year. An all-vs-all BLASTN (e-value < 1 × 10^−5^) was performed using the LTR sequence of 2,825 intact LTR retrotransposons. The LTR retrotransposons were clustered into putative families according to reciprocal blast similarity. Force-directed graph drawings were generated using Cytoscape^53^. The corresponding phylogenetic tree was drawn from each cluster using MEGA 6.0 with a neighbor-joining mode and 1000 bootstrap tests^54^. The solo and truncated LTRs were identified using methods previously described^55^.

### Gene prediction

The strategy for gene prediction in *G. barbadense* genome was to combine several lines of evidence and integrate them using EVM^56^ software to generate the consensus gene model, including *de novo* predictions on the repeat-masked genome (P), protein alignments to genome sequencing (H), transcript alignments to genome sequencing (C). We input the repeat-masked genome to AUGUSTUS^57^, GeneID^58^, GlimmnerHMM^59^, FGENESH^60^ and GenScan^61^. AUGUSTUS, GeneID, GlimmnerHMM and GenScan were used with gene model parameters trained for *Arabidopsis*. FGENESH was used with Dicots plant (*Arabidopsis*) as the parameters. We aligned the protein sequence of five sequenced plants (*Arabidopsis*, Papaya, Grape, Cacao and *Populus*) onto the *G. barbadense* genome using genBlastA^62^ at an *E*-value cutoff of 1 × 10^−5^, and then we extended 1.5 Kb on each side of the homologous genome sequences. The extended genome sequence and protein sequence from genBlastA was processed to get accurate boundaries of exons by GeneWise^63^. The transcript data was processed by PASA^64^ to generate spliced alignments. All of the above resources were combined by EVM^56^ to produce the consensus gene model. After we obtained the consensus gene models, those gene models were filtered which were just supported by only one *de novo* gene prediction method and contained TE family domains. The remaining gene models were updated (annotation of UTR regions and alternative splice events) using PASA^64^.

### Gene annotation

The *G. barbadense* predicted proteins were annotated based on alignment to the TAIR, SwissProt and TrEMBL databases with BLASTP at an E-value cutoff of 1 × 10^−5^, identity ≥ 0.25 and alignment length ≥ 100aa. InterPro was used to annotate motifs and domains by comparison with publicly available databases including Pfam, PRINTS, PROSITE, ProDom and SMART. The pathway in which the gene might be involved was derived from the matched genes in KEGG database^65^. The Gene Ontology information for each gene code was extracted from the InterPro results.

### SNP calling

After filtering reads with low read scores (<20) and accounting for PCR redundancy, the short sequencing data that were mapped to the assembled *G. barbadense* genome and those that were derived from the diploid progenitors (A1, A2, and D5) and from the tetraploid *G. hirsutum* (AD1) were subjected to SNP detection using GATK^66^. The mapping files that were derived from the same diploid genome clade were pooled together for SNP calling using the parameters of “-stand_call_conf 50.0 -stand_emit_conf 10.0 -dcov 500 -ploidy 4”. To remove low quality SNPs, the following parameters were applied (-clusterWindowSize 10 -filter “QD < 2.0” -filterName QualByDepth -filter “MQ < = 40.0” -filterName MapQual -filter “QUAL < 100” -filterName QScore -filter “MQ0 > = 10 && ((MQ0/(1.0 * DP)) > 0.1)” -filterName MapQualRatio -filter “FS > 60.0” -filterName FisherStrandBias).

We used SnpEff^67^ with default parameters to annotate SNPs, using *G. barbadense* as reference. SNPs were categorized according to gene position as intergenic, upstream (within 5 kb of upstream) or downstream (within 5 kb of downstream) and as introns or exons.

### Gene expression analysis by RNA-Seq

Total RNAs of eight different cotton tissues or developmental stages were isolated using a modified guanidine thiocyanate method^68^, and cDNA libraries were constructed and sequenced according to Illumina’s protocols (www.illumina.com). Low quality RNA-Seq reads were removed from raw data using the FASTX-toolkit^69^. High-quality paired-end reads were obtained and mapped to the *G. barbadense* genome assembly using default parameters of TopHat^70^. For gene expression, the FPKM (Fragments Per Kilobase of transcript per Million mapped reads) were calculated using Cufflinks^71^.

### Gene cluster

We used the OrthoMCL^72^ software (version 2.0.3) to classify the complete set of protein-coding genes from the D5 genome (*G. raimondii*^8^) and the At and Dt of *G. barbadense* into narrowly defined gene lineages. Firstly, the all-vs-all BLASTP procedure was conducted using primary protein sequences in these three genome/subgenomes (E-value 1e-5). Then, the result of BLASTP was loaded into a local mysql database. Finally, we used the mcl algorithm to classify them into orthologous clusters (–abc -I 1.5). A customized Perl script was programmed to count gene numbers of each cluster.

### Identification of transcription factor

We applied HMMER (version 3.0)^73^ to identify the gene families of transcription factors (TFs) in the *G. barbadense* genome using the PlnTFDB^74^ to classify the TFs.

### Identification of homoeologous genes

To identify homoeologous genes, we associated genes in At and Dt with those in the D5 reference genome using MCscanX with default settings^75^. After removing the tandem duplications and multiple matches, syntenic blocks containing more than five aligned protein gene pairs were identified; these were regarded as homoeologous gene pairs.

## 

## Additional Information

**Accession codes**: DNA sequence data, assembly, annotation and the browser of the G. barbadense genome are available through our website at http://cotton.cropdb.org. DNA raw data, RNA-seq sequence and genome assembly data has been also deposited at GenBank and the BioProject ID PRJNA219156.

**How to cite this article**: Yuan, D. *et al.* The genome sequence of Sea-Island cotton (*Gossypium barbadense*) provides insights into the allopolyploidization and development of superior spinnable fibres. *Sci. Rep.* **5**, 17662; doi: 10.1038/srep17662 (2015).

## Supplementary Material

Supplementary Information

## Figures and Tables

**Table 1:** Characteristics of the *G. barbadense* genome.

	Whole genome	Allocated to subgenomes		
		At-subgenome	Dt-subgenome	Ungrouped
Assembly				
Scaffold N50 (Mb)	0.260	0.253	0.306	0.157
Maximum scaffold length (Mb)	2.15	1.63	2.15	0.96
Minimum scaffold length (Mb)	0.001	0.001	0.001	0.001
Number of scaffolds	29,751	14,319	6,967	8,465
Total length of assemblies (Mb)	2,573.19	1,493.53	852.98	226.68
Total gaps in assemblies (Mb)	334.55	223.47	82.35	28.72
Annotation				
Number of protein-coding genes	80,876	36,947	34,575	9,354
Average gene density (per 100 kb)	3.14	2.47	4.05	4.12
Average exon/intron sizes (bp)	283.40/423.41	283.07/434.49	281.81/422.92	290.92/449.44
Total size of transposable elements (Mb)	1,778.62	1097.99	541.57	139.06
