# Supplementary material for: The genome sequence of Sea-Island cotton (Gossypium barbadense) provides insights into the allopolyploidization and development of superior spinnable fibres
Source: Sci Rep. 2015 Dec 4;5:17662. doi: 10.1038/srep17662 (PMC4669482; doi:10.1038/srep17662)
Supplement: Supplementary Information [file srep17662-s1.doc]

# Supplementary information

# The genome sequence of Sea-Island cotton (*Gossypium* *barbadense*) provides insights into the allopolyploidization and development of superior spinnable fibres

Daojun Yuan^1,5^, Zhonghui Tang^2,1,5^, Maojun Wang^1,5^, Wenhui Gao^1^, Lili Tu^1^, Xin Jin^1^, Lingling Chen^1,3^, Yonghui He^1^, Lin Zhang^1^, Longfu Zhu^1^, Yang Li^1^, Qiqi Liang^1^, Zhongxu Lin^1^, Xiyan Yang^1^, Nian Liu^1^, Shuangxia Jin^1^, Yang Lei^3^, Yuanhao Ding^1^, Guoliang Li^1,2,3^, Xiaoan Ruan^2,1^, Yijun Ruan^2,1,4,6^ & Xianlong Zhang^1,6^

^1^ National Key Laboratory of Crop Genetic Improvement, Huazhong Agricultural University, Shizishan Street, Wuhan, Hubei 430070, China

^2^ The Jackson Laboratory for Genome Medicine, 10 Discovery Drive, Farmington, CT 06032, USA

^3^ College of Informatics, Huazhong Agricultural University, Shizishan Street, Wuhan, Hubei 430070, China

^4^ Department of Genetics and Developmental Biology, University of Connecticut Health Center, 400 Farmington Ave, Farmington, CT 06032, USA

^5^ These authors contributed equally to this work

^6^ Correspondence should be addressed to X.Z. (xlzhang@mail.hzau.edu.cn) or Y.R. (Yijun.Ruan@jax.org).

Corresponding author: Yijun.Ruan@jax.org and Xianlong Zhang

E-mail: [xlzhang@mail.hzau.edu.cn](mailto:xlzhang@mail.hzau.edu.cn)

Tel: +86-27-87280510

Fax: +86-27-87280196

**Supplementary Figures**


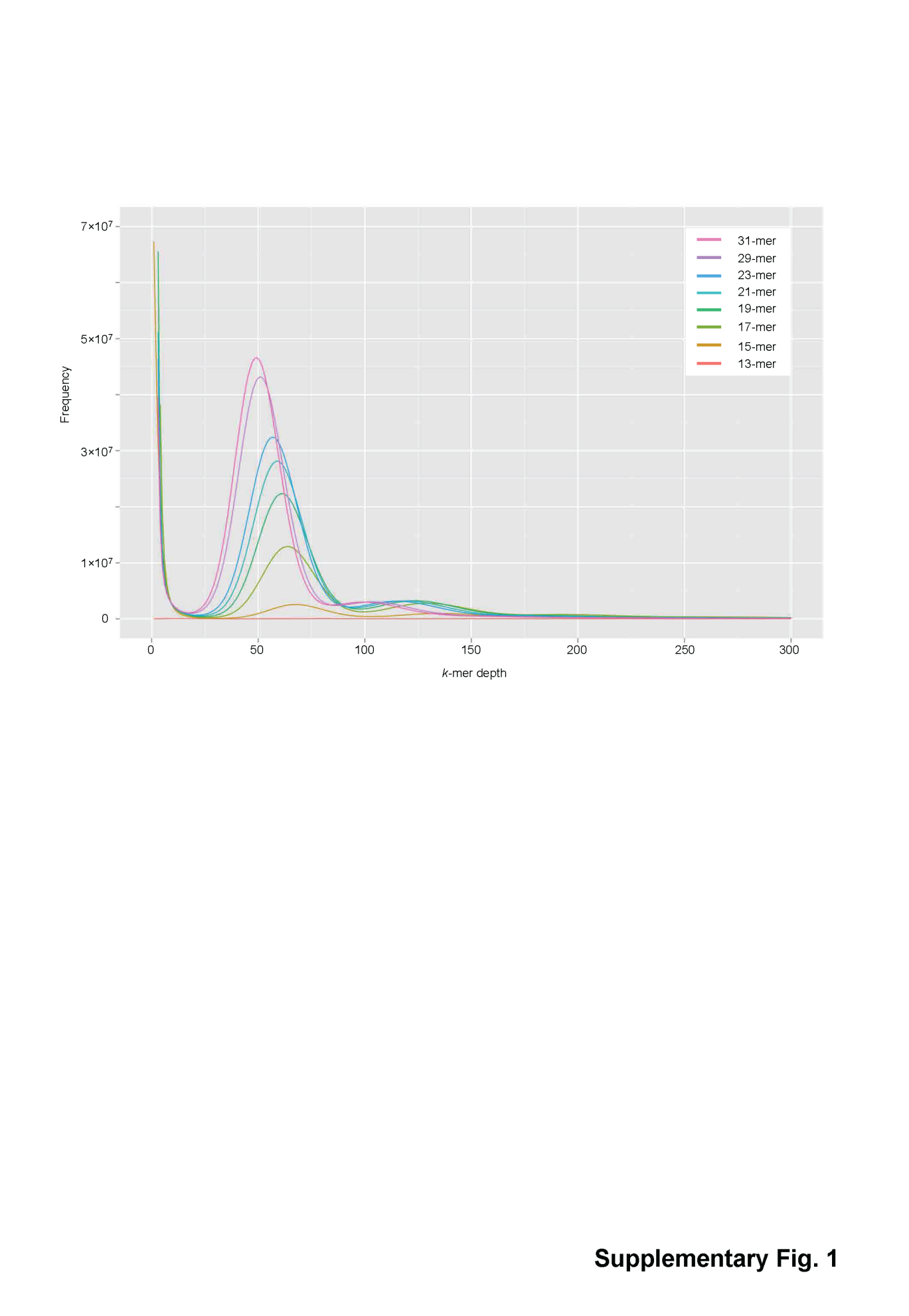


## Supplementary Figure 1 Multiple *k*-mer depth distributions.

Total shotgun sequencing reads were used to calculate *k*-mer depth distribution. Shown are the distributions in *k*-mer size ranges from 13 bp to 31 bp. The 31-mer demonstrated the sharpest distribution and peaked at 48 of the *k*-mer depth. The y-axis represents frequency and the x-axis represents *k*-mer depth.


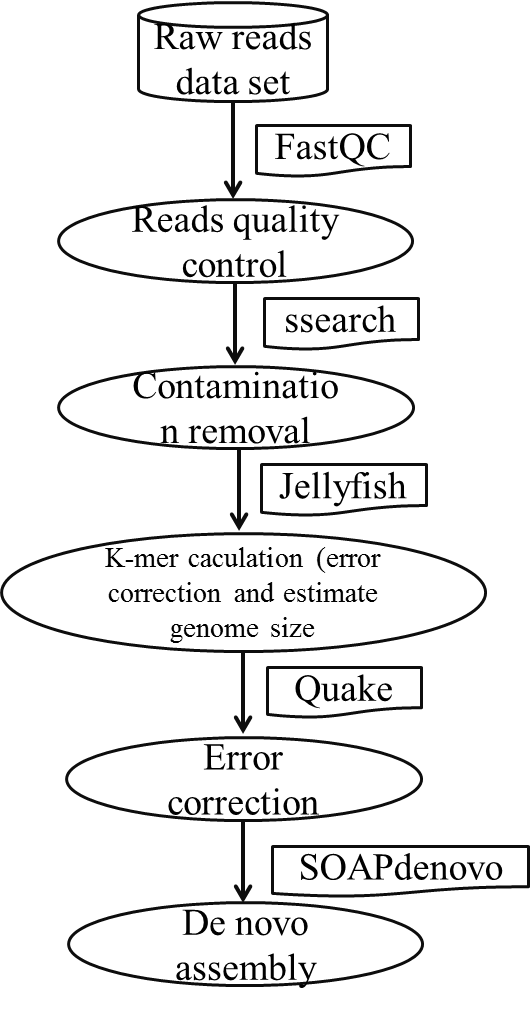


## Supplementary Figure 2 The workflow of *De* *Novo* Assembly.

| 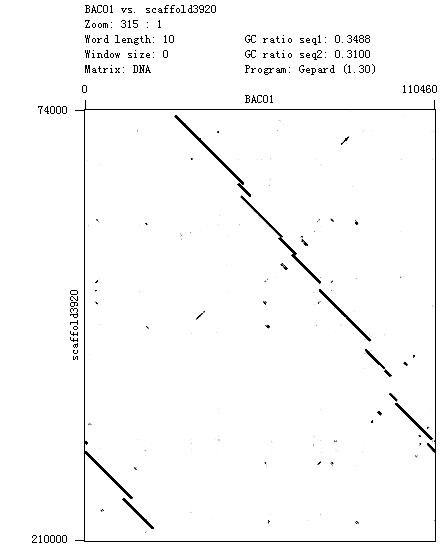 | 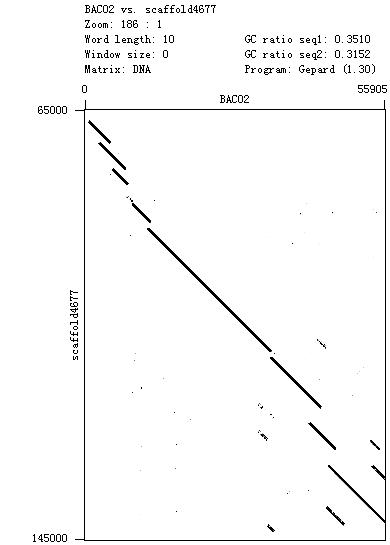 |
| --- | --- |
| 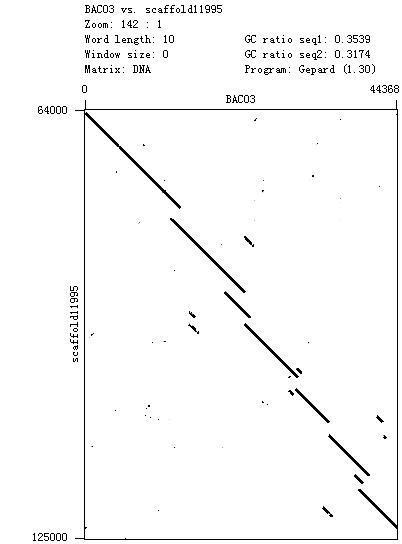 | 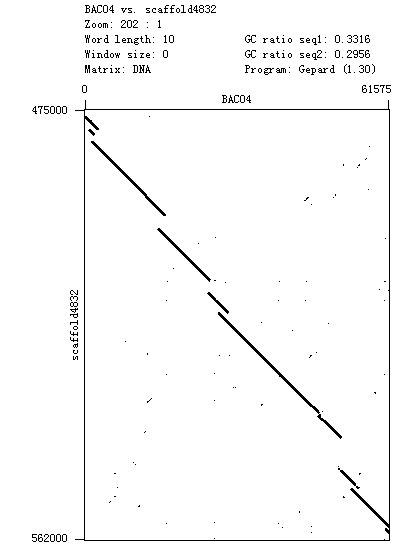 |
| 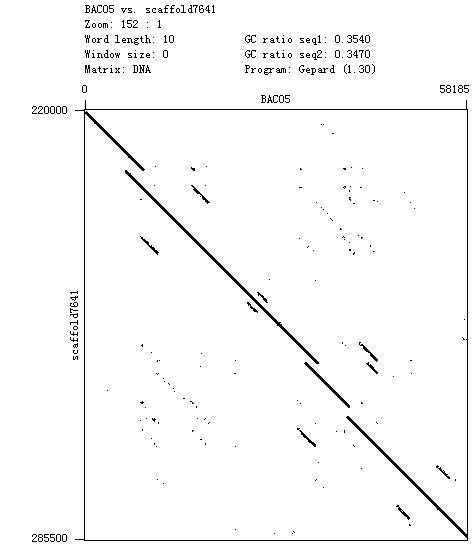 | 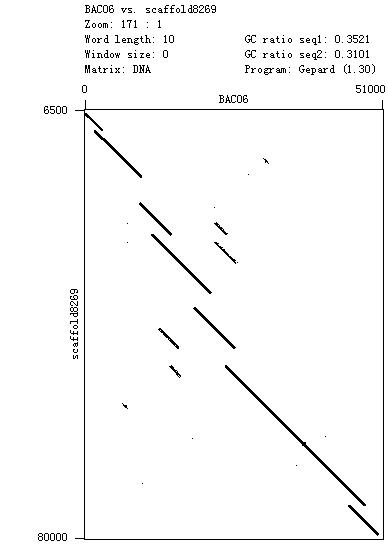 |
| 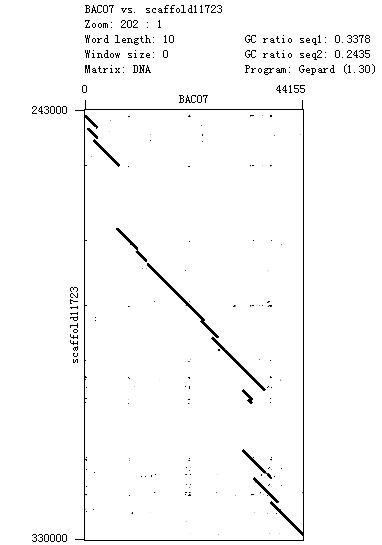 | 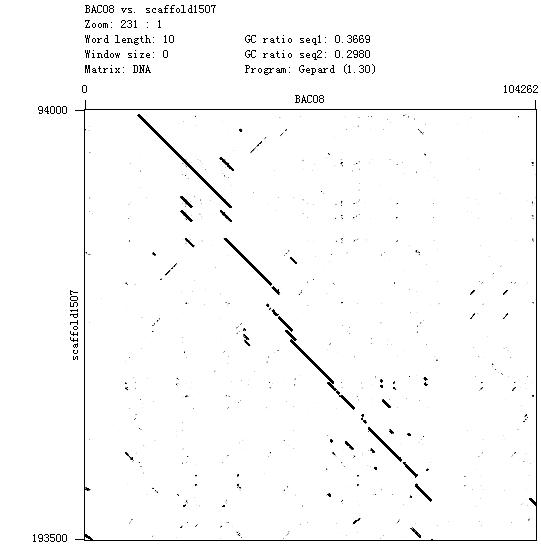 |
| 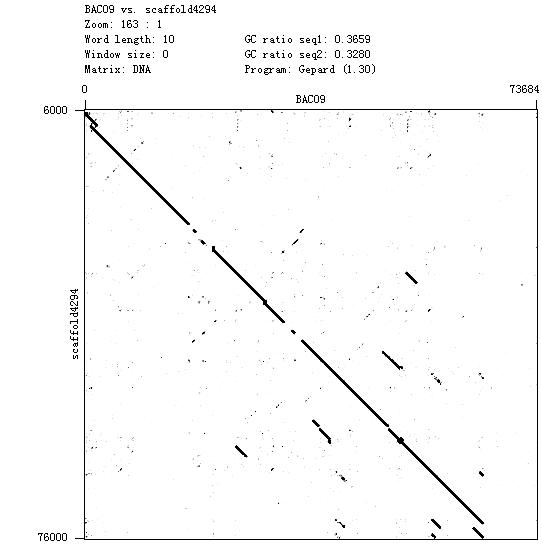 | 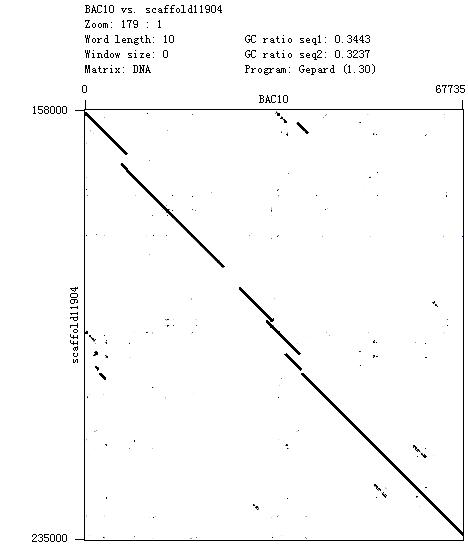 |

## Supplementary Figure 3 Comparison of assembled scaffolds with BAC clone sequences of *G. barbadense*.


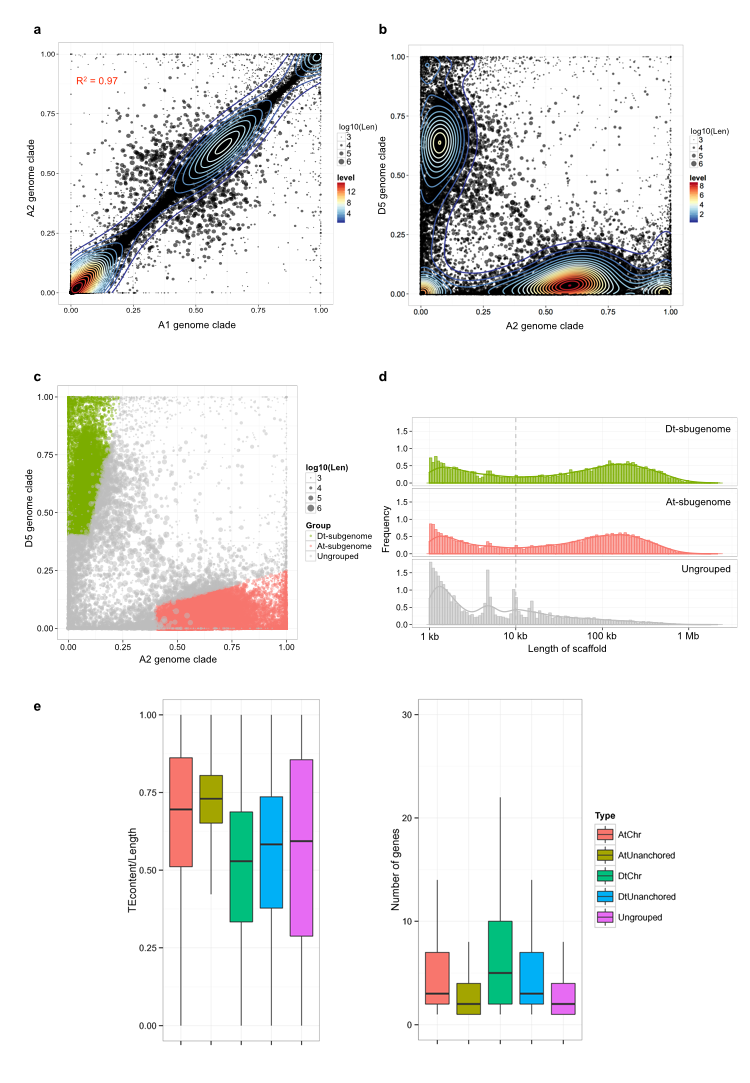


## Supplementary Figure 4 Assignment of assembled scaffolds to subgenomes of *G. barbadense* and characterization of assigned scaffolds (Related to Figure 1).

**a**. The scatter plot with contour density shows the average (%) coverage of the mapped base pairs (0 to1, y- and x-axis) of the diploid A1 and A2 genome clades. Results from the A1 and A2 datasets were highly correlated (R^2^ = 0.97). The size of dot in the plot represents scaffold length and the contour map reflects the density distribution.

**b**. The scatter plot with contour density shows the average (%) coverage of the mapped base pairs of the diploid A2 and D5 genome clades. The contours represent scaffold density in each region in the map.

**c**. The scatter plot shows the average (%) coverage of the mapped base pairs of the diploid A2 and D5 progenitor genome clades. The scaffolds assigned to the At- and D-subgenomes are highlighted in light red and green, respectively.

**d**. The distribution of length of the scaffolds assigned to the A-subgenome (top), the D-subgenome (middle) and those that were “ungrouped” (bottom).

**e**. Box plots show the TE ratio, gene number and gene density in categorized scaffolds. Column 1: Scaffolds assigned to the A-subgenome anchored to the D5 reference genome; 2: Scaffolds assigned to the A-subgenome but not anchored to the D5 reference genome; 3: Scaffolds assigned to the D-subgenome anchored to the D5 reference genome; 4: Scaffolds assigned to D-subgenome but not anchored to the D5 reference genome; 5: Ungrouped scaffolds.


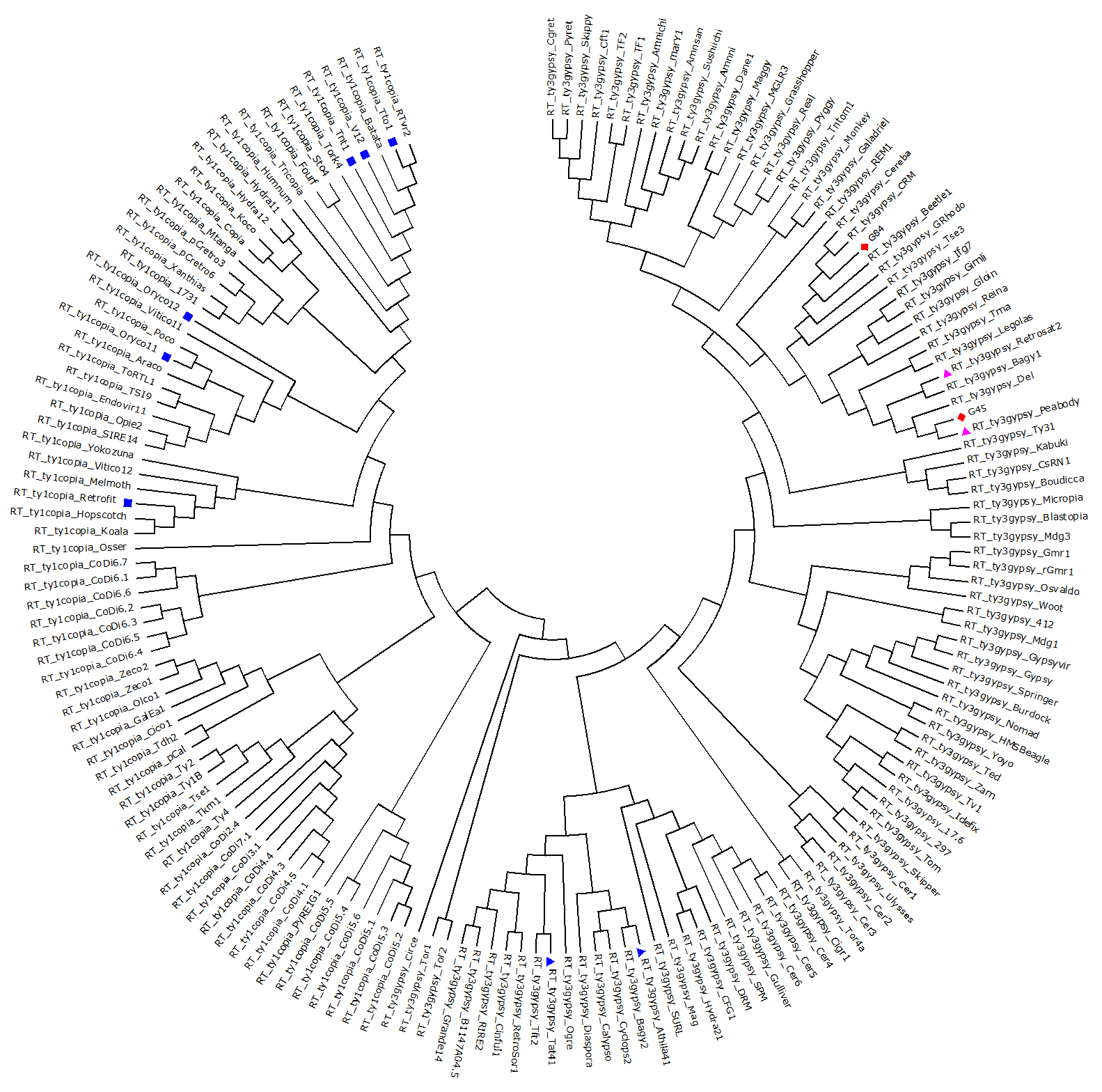


## Supplementary Figure 5 Phylogenetic tree of RT domain for LTR.

**
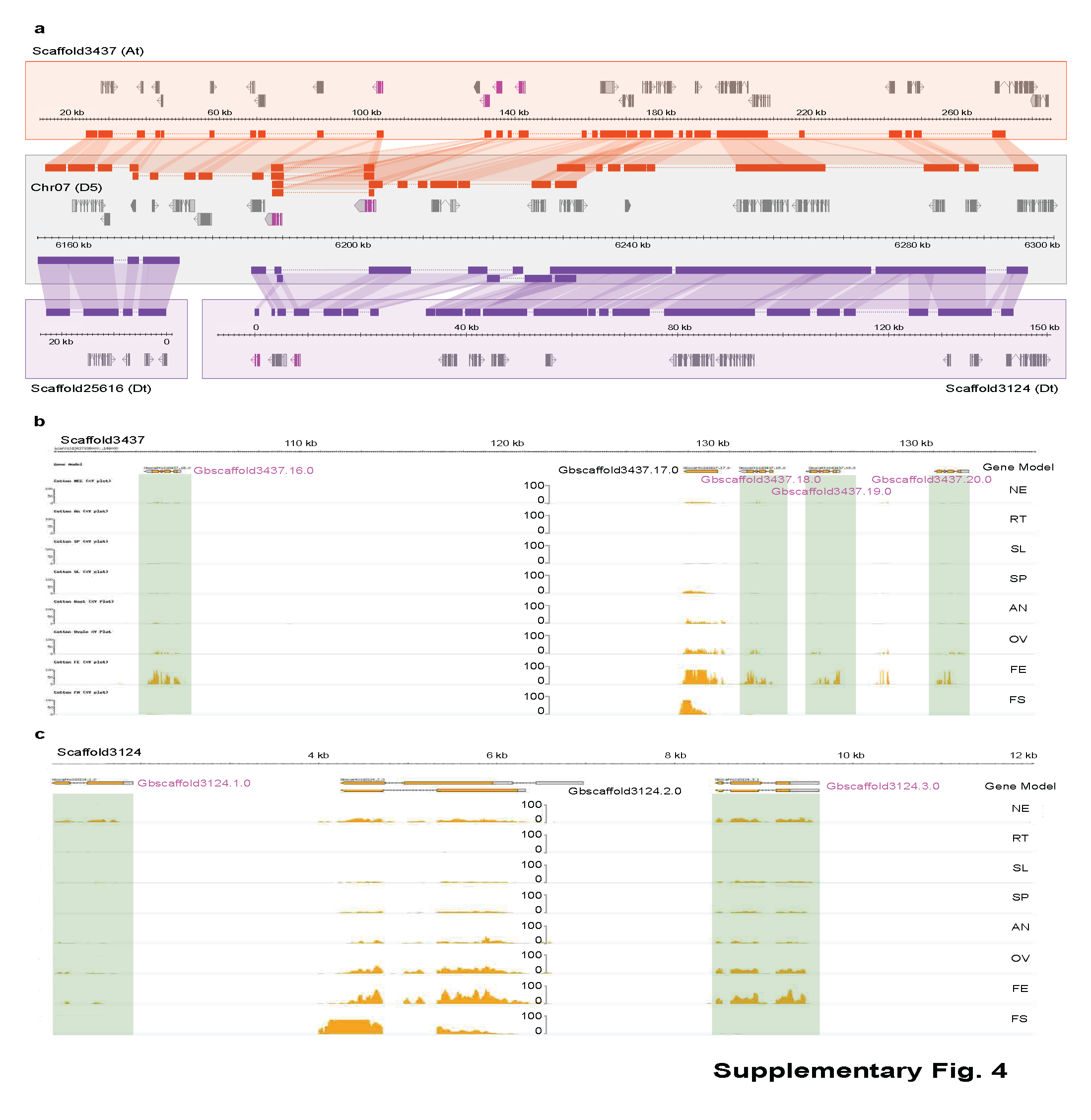
**

## Supplementary Figure 6 An example of the micro-syntenic relationship between the subgenomes of *G. barbadense* and the D5 reference genome.

**a**. Micro-synteny block of the genomic region surrounding the tandem duplication of HD-Zip genes in the At-subgenome and its collinear regions in the Dt-subgenome and D5 genome. The HD-Zip genes in this region are highlighted with magenta. The red blocks connect regions conserved in the At-subgenome and in the D5 genome, while the purple blocks connect regions conserved in the Dt-subgenome and in the D5 genome.

**b**. The screenshot shows RNA-Seq mapping of the four HD-Zip genes of the At-subgenome in this syntenic block. The gene IDs of HD-Zip are highlighted with magenta.

**c**. The screenshot shows RNA-Seq mapping of two HD-Zip genes of Dt-subgenome in syntenic block. The names of HD-Zip genes are highlighted in magenta. RNA-Seq data are from the NE, RT, SL, SP, AN, OV, FE and FS stages. All RNA-Seq tracks are adjusted to the same scale with max at 100. The HD-Zip gene regions and their RNA-Seq mapping are highlighted in light green.


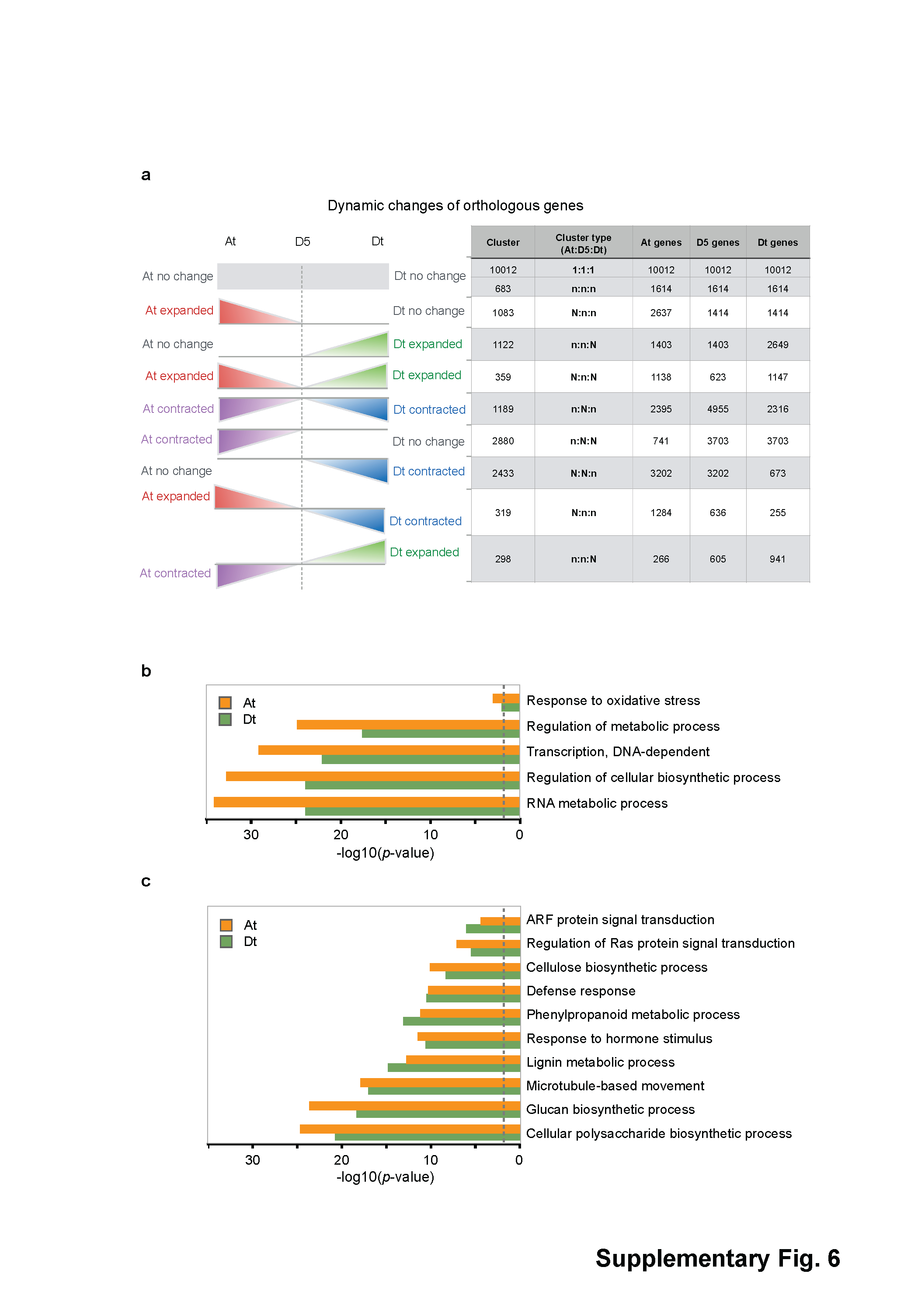


## Supplementary Figure 7 Dynamic changes in orthologous genes in the At- and Dt- subgenomes of *G. barbadense* (related to Figure 4).

**a**. Using the D5 genome (*G. raimondii*) as a reference, the orthologous gene clusters were categorized into nine groups, according to the expansion or contraction of the orthologous gene family in the two subgenomes. The left panel shows the categories changes that occurred in these orthologous gene families. The right panel shows the orthologous gene cluster numbers and gene numbers involved in each category. “N” indicates numbers larger relative to “n.”

**b**. Gene Ontology (GO) enrichment analysis of genes present in the category of 1:1:1 orthologous clusters.

**c.** GO enrichment analysis of orthologous genes involved in duplication events in D5 and subgenomes. Bonferroni corrected *p*-values were transformed by -log10.

**
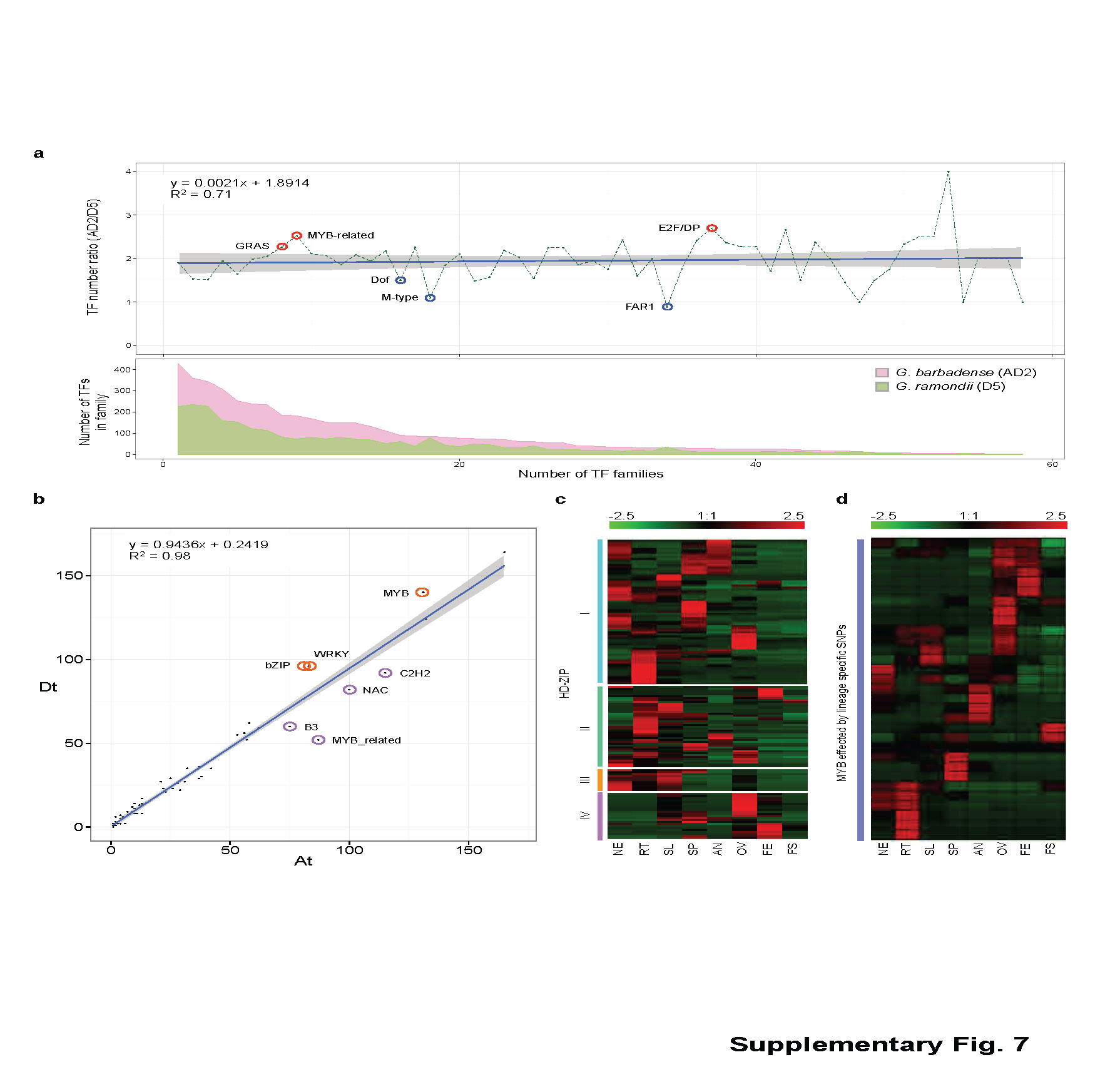
**

## Supplementary Figure 8 TF families in *G. barbadense* genome.

**a**. Comparing the number of TF families in the *G. barbadense* (AD2) and *G.* *raimondii* (D5) genomes. In the upper panel, the dotted line plot shows the ratio of the TFs in the *G. barbadense* and *G.* *raimondii* genomes. The solid blue line represents the linear regression of the TF ratio and the gray shade represents the confidence interval. Using the *G.* *raimondii* genome (D5) as the reference, the expanded (red) and contracted (blue) TF families are highlighted with circles. In the lower panel, the plot shows the number of TF in each TF families of *G. barbadense* and *G.* *raimondii* genomes.

**b**. Comparing the number of TF families in the At- and Dt- subgenomes in *G. barbadense*. The changed TF families are highlighted with circles. Red circles indicate the TF families that expanded in the Dt-subgenome, purple circles indicate expanded TF families in the At-subgenome.

**c**. Heatmap shows the expression profiles of HD-ZIP gene family in groups of phylogenetic clades. Related to **Figure 4c**.

**d**. Heatmap shows the expression profiles of MYB genes that carry non-synonymous lineage-specific SNPs in the At-subgenome of *G. barbadense*.


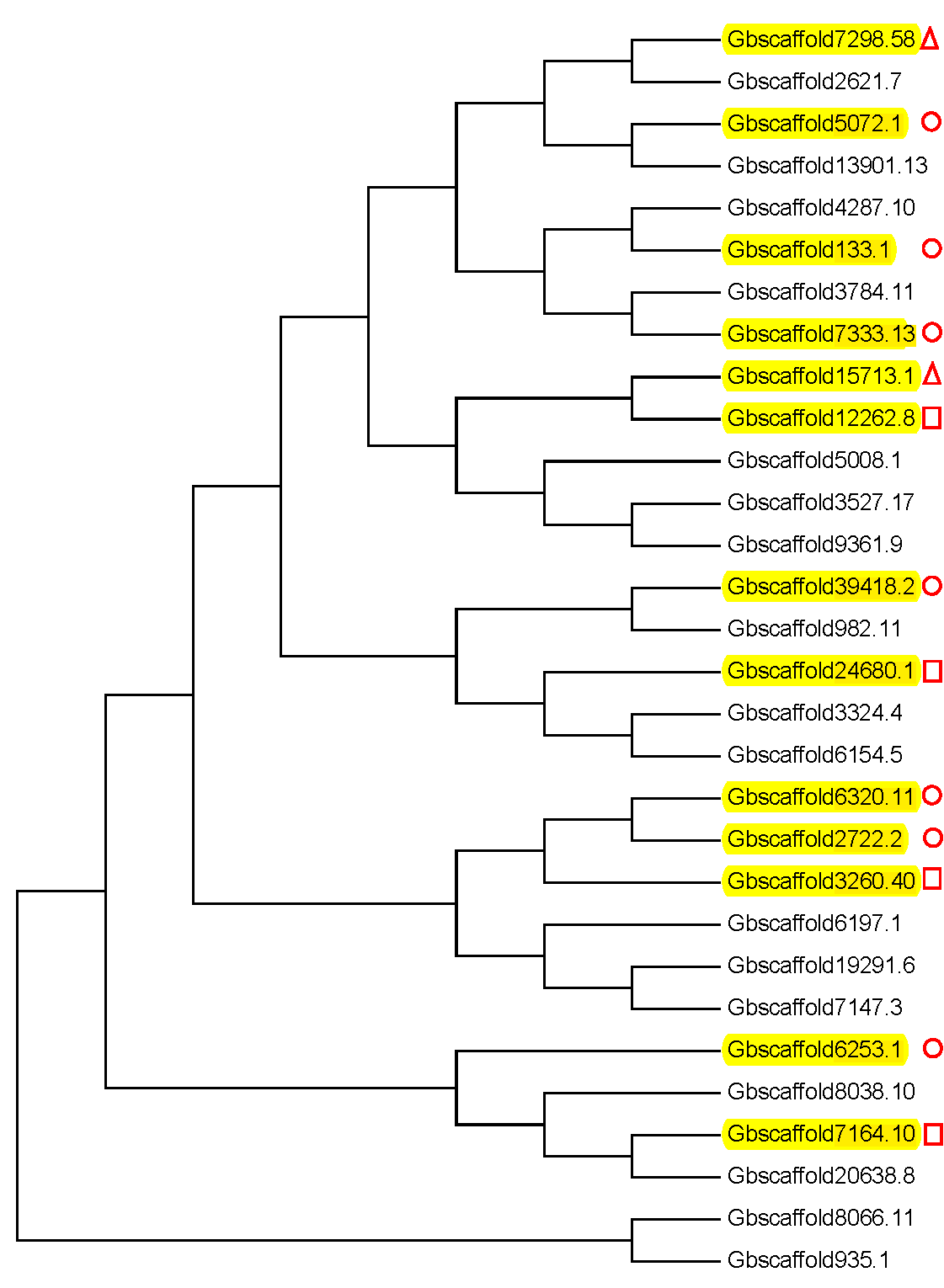


## Supplementary Figure 9 Phylogenetic tree of the promoter of 31 MYB genes.

The 2000 bp upstream sequences of the 31 MYB genes were extracted. MEGA 6.0 was used to align the 31 sequences and construct the Neighbor-Joining (NJ) tree with the bootstrap of 1000. The circle, rectangle and triangle represent the gene expressed preferentially in ovule, fibre elongation stage and fibre secondary cell wall synthase stage respectively.


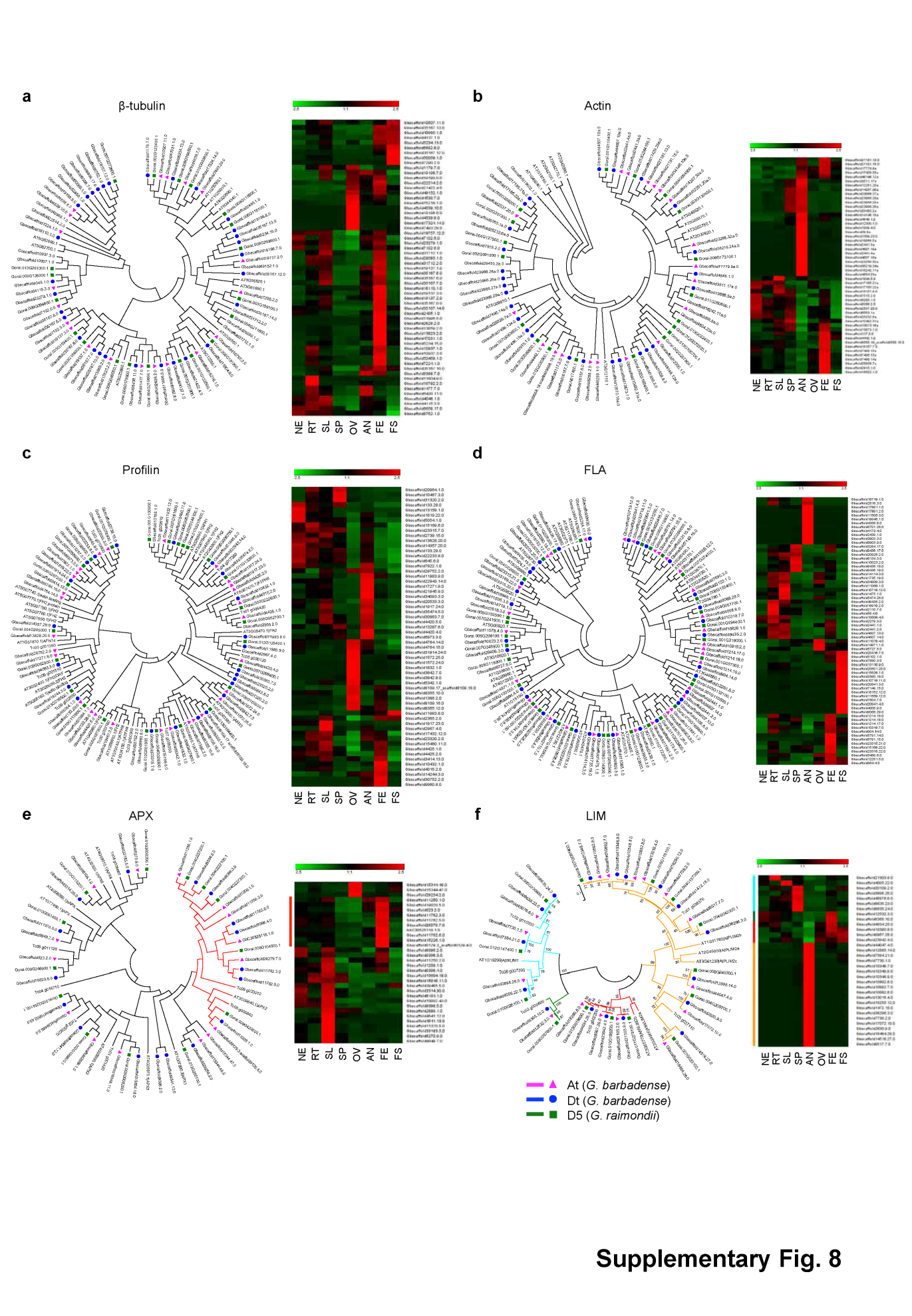


## Supplementary Figure 10 Phylogenetic and expression profile analysis of genes potentially involved in fibre development.

Phylogenetic trees (left) and gene expression profiles (right) for the following gene families: β-tubulin (a), actin (b), profilin (c), FLA (d), APX (e) and LIM (f). The expression profile in (e) is specifically marked for the phylogenetic clade in red. The expression profile in (f) is marked for each of the phylogenetic clades with corresponding color code. In each phylogenetic tree, the genes of the At-subgenome are denoted with red triangles. Genes of the Dt-subgenome are denoted with blue circles. Genes of the D5 genome (*G.* *raimondii*) are denoted with green rectangles. Genes from *Arabidopsis* (prefix of gene name is At) are included in phylogenetic tree as an out-group. The heatmap columns represent RNA-Seq data from NE, RT, SL, SP, AN, OV, FE and FS stages.

**
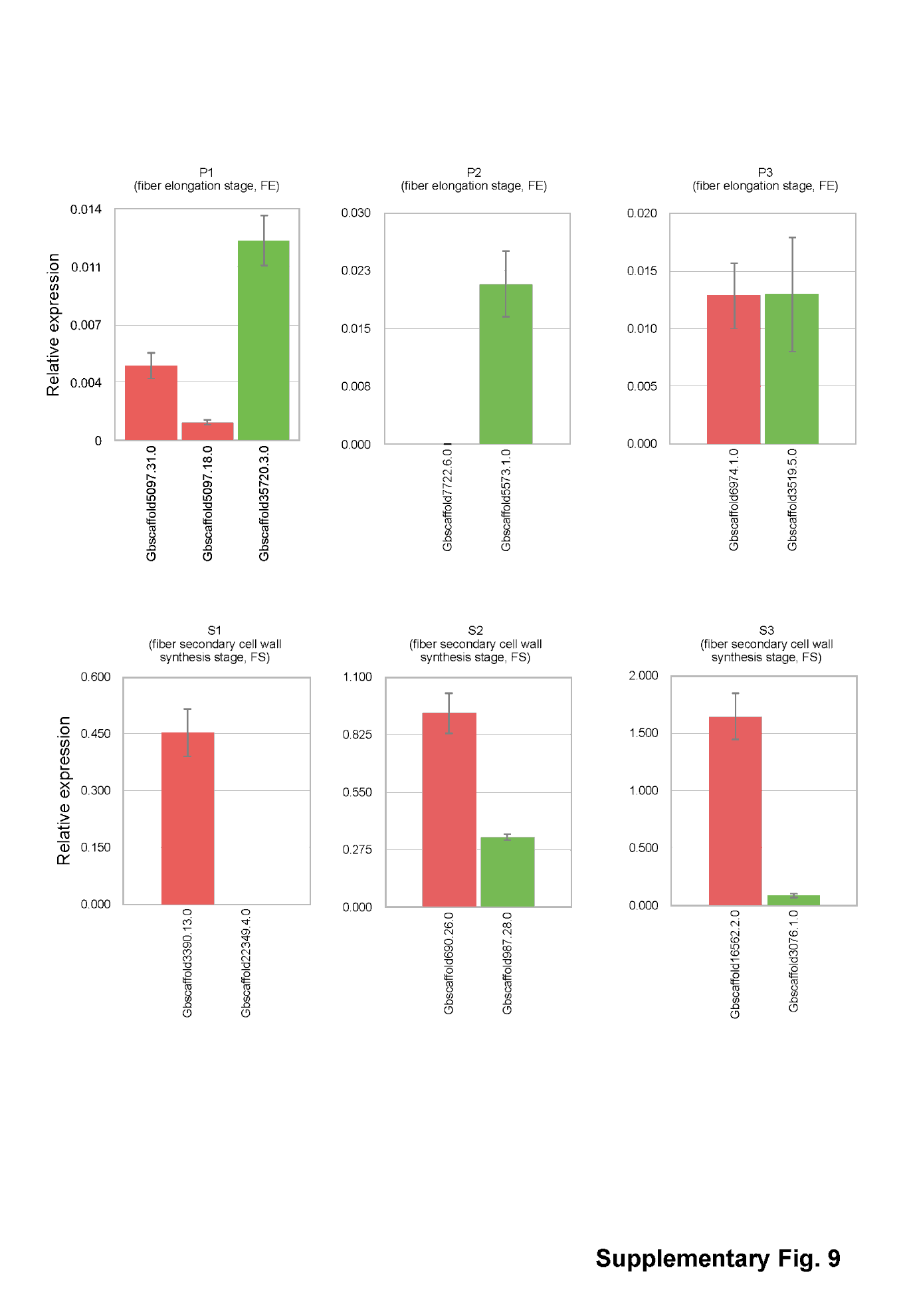
**

## Supplementary Figure 11 qRT-PCR experiments validated the expressions of CesA genes.

The expression profiles of CesA genes belonging to five CesA gene clades were analyzed by qRT-PCR with 40 PCR amplification cycles. The CesA genes from At- and Dt- subgenomes are donated with red and green colors in the bar plots, respectively. The fibre tissue of *G. barbadense* representing fibre elongation stage was harvested at 10 DPA, while the fibre tissue of *G. barbadense* representing fibre secondary cell wall synthesis stage was harvested at 20 DPA. The transcription levels of each gene were normalized with the expression level of housekeeping gene Ubiquitin 7. The qRT-PCR was performed four biology replicated and three technical replicates for each gene. Error bars in the bar plots denoted standard deviation of triplicates. All qRT-PCR primers were shown in the **Supplementary Table 19**.

**Supplementary Tables**

***Supplementary Table 1. Summary of G. barbadense genome sequencing.***

| **Library insertion size** | **Lane** | **Read length** | **Raw data (Gb)** | **Usable data (Gb)** | **Base-pair coverage*** | **Physical coverage*** |
| --- | --- | --- | --- | --- | --- | --- |
| ~ 500 bp | 7 | 100 bp × 2 | 247.54 | 245.81 | 95.28 | 237.58 |
| ~ 5 kb | 3 | 75 bp × 2 | 85.68 | 76.25 | 29.55 | 985.15 |
| ~ 10 kb | 3 | 75 bp × 2 | 83.05 | 70.14 | 27.19 | 1812.53 |
| ~ 20 kb | 2 | 75 bp × 2 | 54.66 | 54.38 | 21.08 | 2810.53 |
| **Total** | 15 |  | 470.93 | 446.58 | 173.1 | 5845.79 |

*The estimated genome size is 2.57 Gb (**Supplementary Figure 1**)

**Supplementary Table 2. Summary of the sequence alignment of BAC clones against published cotton genomes.**

| query | Coverage_Gb | Identity_Gb | Coverage_Ga | Identity_Ga | Coverage_CGI | Identity_CGI | Coverage_JGI | Identity_JGI |
| --- | --- | --- | --- | --- | --- | --- | --- | --- |
| gi_114158934_AC190656.1 | 100.00% | 96.44% | 22.60% | 87.30% | 100.00% | 99.75% | 100.00% | 99.97% |
| gi_112984856_AC188035.3 | 98.88% | 99.14% | 64.17% | 92.08% | 99.03% | 99.93% | 99.83% | 99.94% |
| gi_115312511_AC190814.2 | 96.08% | 96.93% | 18.19% | 87.49% | 99.69% | 99.73% | 100.00% | 100.00% |
| gi_113206022_AC190312.1 | 95.85% | 97.97% | 19.80% | 82.46% | 96.97% | 99.87% | 98.52% | 100.00% |
| gi_112422252_AC187202.3 | 95.43% | 96.53% | 26.73% | 84.34% | 95.67% | 99.89% | 97.39% | 99.99% |
| gi_118601197_AC193940.1 | 95.20% | 97.13% | 32.00% | 90.92% | 97.66% | 99.73% | 99.03% | 99.17% |
| gi_112422289_AC187470.3 | 93.91% | 97.44% | 31.76% | 91.10% | 83.92% | 99.84% | 100.00% | 99.99% |
| gi_111186098_AC187794.3 | 91.43% | 97.30% | 32.78% | 92.29% | 98.51% | 99.84% | 99.64% | 99.99% |
| gi_112984883_AC188028.2 | 91.01% | 96.80% | 15.34% | 92.15% | 99.31% | 99.64% | 100.00% | 99.97% |
| gi_113195782_AC189748.2 | 84.51% | 97.21% | 82.73% | 92.05% | 99.24% | 99.81% | 100.00% | 99.99% |
| gi_117962369_AC193517.1 | 83.11% | 97.53% | 39.52% | 91.83% | 95.31% | 99.95% | 97.30% | 99.96% |
| gi_114159094_AC190815.1 | 82.95% | 97.45% | 53.01% | 92.31% | 84.28% | 99.88% | 99.71% | 99.94% |
| gi_112422287_AC187472.3 | 82.34% | 97.55% | 15.78% | 85.43% | 99.32% | 98.37% | 100.00% | 97.99% |
| gi_117962250_AC193514.1 | 81.18% | 96.96% | 17.48% | 82.90% | 92.33% | 99.82% | 100.00% | 99.98% |
| gi_108860742_AC187396.1 | 80.94% | 96.28% | 36.94% | 90.61% | 73.78% | 99.92% | 99.34% | 99.98% |
| gi_111186085_AC187549.3 | 79.18% | 97.83% | 55.32% | 91.81% | 92.32% | 99.87% | 99.24% | 99.97% |
| gi_114703874_AC188140.2 | 77.91% | 98.19% | 75.59% | 94.13% | 99.03% | 99.80% | 100.00% | 100.00% |
| gi_145864551_AC202825.1 | 74.98% | 86.06% | 74.98% | 84.01% | 74.98% | 83.49% | 74.98% | 83.20% |
| gi_111186102_AC187214.3 | 74.83% | 96.51% | 44.29% | 88.95% | 94.96% | 99.95% | 97.36% | 99.97% |
| gi_111548777_AC187812.3 | 72.51% | 97.71% | 18.25% | 93.26% | 88.23% | 99.84% | 99.57% | 99.87% |
| gi_112984849_AC190263.1 | 72.34% | 97.49% | 27.40% | 92.74% | 97.11% | 99.81% | 96.23% | 99.87% |
| gi_112984882_AC188026.2 | 68.39% | 97.59% | 25.30% | 86.59% | 72.92% | 99.98% | 72.92% | 99.99% |
| gi_112422244_AC187221.3 | 67.93% | 97.78% | 23.46% | 90.87% | 97.45% | 99.77% | 100.00% | 99.98% |
| gi_114703876_AC188401.2 | 67.07% | 98.04% | 50.23% | 92.62% | 61.41% | 99.62% | 93.52% | 99.38% |
| gi_114703763_AC188199.2 | 63.21% | 97.23% | 17.26% | 85.26% | 84.82% | 99.91% | 99.11% | 99.98% |
| gi_115292505_AC190805.2 | 60.96% | 97.46% | 50.32% | 92.57% | 62.14% | 99.96% | 62.14% | 99.96% |
| gi_115292545_AC188016.2 | 60.32% | 97.66% | 12.71% | 81.74% | 98.02% | 99.72% | 98.98% | 99.90% |
| gi_124001805_AC197182.1 | 58.94% | 97.82% | 48.07% | 94.23% | 85.47% | 99.79% | 86.26% | 99.94% |
| gi_114703948_AC188037.2 | 57.99% | 96.96% | 28.15% | 85.17% | 77.49% | 99.98% | 79.39% | 99.64% |
| gi_113195783_AC190279.1 | 57.83% | 98.63% | 13.93% | 88.70% | 60.04% | 99.95% | 60.04% | 99.98% |
| gi_112984831_AC188142.2 | 57.54% | 97.68% | 20.58% | 93.40% | 57.54% | 99.33% | 58.36% | 97.88% |
| gi_118601249_AC193992.1 | 57.53% | 96.96% | 32.16% | 92.66% | 57.67% | 99.88% | 57.67% | 99.99% |
| gi_118601251_AC193994.1 | 57.21% | 97.76% | 44.79% | 94.46% | 70.40% | 99.83% | 70.43% | 99.93% |
| gi_110797545_AC187473.2 | 56.62% | 97.19% | 19.74% | 88.69% | 64.84% | 99.82% | 83.04% | 96.99% |
| gi_111186084_AC187545.3 | 52.30% | 97.47% | 29.80% | 94.29% | 81.21% | 96.70% | 72.73% | 99.89% |
| gi_118601573_AC194316.1 | 51.49% | 96.90% | 13.83% | 85.57% | 48.03% | 99.80% | 71.67% | 96.19% |
| gi_124001817_AC197194.1 | 51.46% | 97.97% | 32.67% | 93.14% | 60.42% | 98.98% | 68.71% | 96.58% |
| gi_114703853_AC188200.2 | 51.39% | 92.62% | 23.71% | 90.47% | 96.24% | 99.30% | 97.08% | 99.83% |
| gi_110625276_AC187200.2 | 51.19% | 96.93% | 22.79% | 88.39% | 98.15% | 99.95% | 98.30% | 99.99% |
| gi_114329144_AC187201.3 | 50.79% | 97.61% | 15.55% | 83.36% | 99.86% | 99.94% | 100.00% | 100.00% |
| gi_111186077_AC187578.3 | 50.27% | 97.30% | 24.83% | 93.66% | 59.88% | 99.80% | 71.65% | 97.07% |
| gi_145864543_AC202817.1 | 50.06% | 97.66% | 11.66% | 91.26% | 74.67% | 99.73% | 75.71% | 99.95% |
| gi_110625258_AC187220.2 | 49.64% | 96.94% | 21.00% | 83.67% | 54.09% | 99.74% | 57.66% | 99.99% |
| gi_113195781_AC188842.2 | 49.14% | 98.09% | 51.23% | 92.58% | 95.00% | 99.75% | 99.47% | 100.00% |
| gi_145864558_AC202831.1 | 49.12% | 97.05% | 23.55% | 90.37% | 73.05% | 99.94% | 73.05% | 99.95% |
| gi_111186076_AC187136.3 | 48.86% | 96.88% | 10.77% | 82.83% | 39.94% | 99.38% | 79.24% | 99.23% |
| gi_112984841_AC187836.2 | 48.82% | 97.28% | 17.52% | 93.39% | 79.11% | 99.96% | 78.08% | 99.18% |
| gi_112984839_AC190260.1 | 47.93% | 97.52% | 46.51% | 93.29% | 59.54% | 99.79% | 52.75% | 99.91% |
| gi_117961521_AC193506.1 | 46.82% | 96.68% | 14.17% | 91.99% | 63.21% | 99.81% | 79.40% | 97.07% |
| gi_110625249_AC187225.2 | 46.78% | 97.62% | 20.10% | 93.54% | 95.61% | 99.29% | 99.71% | 99.97% |
| gi_109690001_AC188017.1 | 46.65% | 97.49% | 75.17% | 92.38% | 94.35% | 99.75% | 94.89% | 99.96% |
| gi_111186083_AC187471.3 | 46.58% | 96.42% | 51.30% | 90.58% | 47.44% | 99.66% | 58.29% | 98.04% |
| gi_118601583_AC194326.1 | 46.41% | 95.52% | 14.42% | 85.88% | 46.66% | 99.32% | 93.63% | 99.72% |
| gi_145864547_AC202821.1 | 45.22% | 96.74% | 17.82% | 90.94% | 45.17% | 99.77% | 46.57% | 99.90% |
| gi_111186082_AC187546.3 | 43.00% | 99.19% | 32.75% | 95.59% | 54.95% | 99.92% | 55.41% | 99.98% |
| gi_111548766_AC187848.3 | 42.73% | 95.50% | 57.60% | 94.16% | 91.68% | 99.50% | 95.02% | 99.98% |
| gi_113195780_AC187838.3 | 42.00% | 97.28% | 7.97% | 88.82% | 98.71% | 99.68% | 96.97% | 99.66% |
| gi_114431705_AC185509.3 | 40.89% | 95.98% | 12.04% | 93.20% | 35.83% | 98.03% | 59.58% | 99.29% |
| gi_110797483_AC188018.2 | 40.60% | 97.24% | 17.49% | 94.48% | 60.63% | 99.65% | 61.83% | 99.94% |
| gi_110665852_AC187478.2 | 39.74% | 97.28% | 27.90% | 93.34% | 41.27% | 99.77% | 42.01% | 99.84% |
| gi_110626249_AC187141.2 | 39.69% | 97.18% | 23.94% | 86.62% | 83.12% | 99.77% | 87.51% | 99.54% |
| gi_114703951_AC188760.2 | 38.41% | 97.45% | 62.92% | 92.09% | 98.44% | 99.74% | 100.00% | 99.97% |
| gi_117959276_AC193383.1 | 38.27% | 97.86% | 37.62% | 93.18% | 52.13% | 99.99% | 56.11% | 99.99% |
| gi_114159119_AC190840.1 | 38.11% | 97.87% | 28.81% | 89.16% | 67.18% | 99.71% | 49.20% | 99.43% |
| gi_115361364_AC188840.2 | 37.04% | 98.73% | 32.32% | 91.84% | 97.13% | 99.58% | 100.00% | 100.00% |
| gi_124001815_AC197192.1 | 35.56% | 97.51% | 15.61% | 81.96% | 50.45% | 99.99% | 52.09% | 99.97% |
| gi_115312502_AC190777.2 | 33.85% | 89.58% | 23.99% | 90.13% | 8.74% | 81.14% | 74.35% | 99.14% |
| gi_111548775_AC187550.3 | 33.66% | 96.97% | 24.44% | 90.73% | 33.66% | 99.93% | 39.10% | 98.49% |
| gi_124001813_AC197190.1 | 33.45% | 97.39% | 29.06% | 89.92% | 41.73% | 99.43% | 43.92% | 99.98% |
| gi_112422286_AC187031.3 | 32.39% | 97.83% | 31.75% | 93.01% | 49.60% | 99.88% | 52.59% | 99.22% |
| gi_118200539_AC193751.1 | 32.31% | 97.92% | 16.75% | 90.57% | 32.30% | 99.57% | 39.70% | 98.46% |
| gi_114703882_AC188141.3 | 32.18% | 96.49% | 22.30% | 87.65% | 35.88% | 99.43% | 59.13% | 99.04% |
| gi_145864555_AC202828.1 | 30.74% | 96.72% | 12.43% | 85.36% | 59.53% | 99.92% | 58.97% | 99.93% |
| gi_111186094_AC187801.3 | 29.99% | 94.60% | 26.19% | 90.00% | 83.71% | 98.73% | 77.31% | 95.56% |
| gi_114159115_AC190836.1 | 29.43% | 98.44% | 25.52% | 93.05% | 41.64% | 99.89% | 41.13% | 99.97% |
| gi_114158941_AC190663.1 | 29.37% | 96.01% | 13.41% | 83.21% | 18.18% | 99.58% | 36.87% | 94.24% |
| gi_112984850_AC187882.3 | 29.23% | 97.89% | 16.52% | 94.84% | 41.94% | 99.81% | 44.27% | 99.60% |
| gi_145864546_AC202820.1 | 29.22% | 98.55% | 13.84% | 82.16% | 29.03% | 99.54% | 21.98% | 91.13% |
| gi_111548776_AC187810.3 | 26.59% | 95.13% | 28.55% | 88.58% | 83.30% | 99.64% | 91.37% | 99.98% |
| gi_118601611_AC194354.1 | 26.54% | 98.71% | 34.71% | 90.02% | 26.54% | 99.99% | 26.54% | 100.00% |
| gi_118601576_AC194319.1 | 26.14% | 95.58% | 4.16% | 83.18% | 11.47% | 86.76% | 81.01% | 99.54% |
| gi_145864557_AC202830.1 | 25.70% | 96.33% | 47.17% | 87.43% | 69.12% | 99.71% | 59.64% | 98.12% |
| gi_117961512_AC193505.1 | 23.06% | 96.42% | 22.04% | 94.93% | 51.65% | 99.38% | 53.96% | 98.83% |
| gi_115292491_AC189743.2 | 22.61% | 97.13% | 22.83% | 85.40% | 95.28% | 99.39% | 88.62% | 99.96% |
| gi_145864548_AC202822.1 | 22.51% | 97.65% | 20.04% | 93.88% | 33.84% | 99.13% | 35.71% | 99.76% |
| gi_114158926_AC190648.1 | 22.14% | 97.13% | 22.43% | 87.01% | 42.61% | 99.73% | 34.94% | 99.26% |
| gi_124001816_AC197193.1 | 22.13% | 97.64% | 27.92% | 85.88% | 28.67% | 97.24% | 41.69% | 91.59% |
| gi_145864554_AC202827.1 | 21.79% | 97.53% | 31.10% | 85.52% | 26.12% | 99.88% | 26.12% | 99.91% |
| gi_111186086_AC187548.3 | 21.23% | 97.42% | 13.25% | 84.31% | 81.52% | 99.84% | 81.98% | 99.99% |
| gi_118601605_AC194348.1 | 20.48% | 98.61% | 11.84% | 83.56% | 24.34% | 99.53% | 28.89% | 93.83% |
| gi_145864545_AC202819.1 | 20.41% | 97.48% | 10.78% | 86.83% | 13.59% | 98.39% | 31.07% | 93.44% |
| gi_110625433_AC187213.2 | 20.07% | 91.97% | 15.30% | 90.44% | 97.13% | 99.80% | 99.98% | 99.91% |
| gi_117960686_AC193495.1 | 19.94% | 97.72% | 23.36% | 86.68% | 42.60% | 98.58% | 66.62% | 93.76% |
| gi_145864549_AC202823.1 | 19.85% | 97.88% | 19.71% | 92.68% | 19.85% | 99.84% | 21.14% | 93.71% |
| gi_124001804_AC197181.1 | 18.65% | 97.87% | 21.71% | 90.71% | 20.23% | 99.56% | 22.39% | 98.73% |
| gi_118601640_AC194383.1 | 17.43% | 94.64% | 19.65% | 89.45% | 29.98% | 99.25% | 35.18% | 94.77% |
| gi_112984851_AC188030.3 | 16.34% | 97.10% | 14.81% | 85.62% | 82.73% | 88.28% | 82.73% | 87.20% |
| gi_117959109_AC193321.1 | 15.90% | 95.87% | 22.43% | 84.54% | 55.11% | 98.96% | 44.07% | 98.54% |
| gi_124001810_AC197187.1 | 15.22% | 93.11% | 2.61% | 80.31% | 14.15% | 88.22% | 24.80% | 97.31% |
| gi_118601621_AC194364.1 | 14.83% | 92.56% | 14.08% | 93.11% | 16.93% | 99.83% | 22.16% | 94.37% |
| gi_145864553_AC202826.1 | 13.96% | 95.76% | 8.85% | 91.81% | 16.62% | 99.32% | 17.02% | 99.60% |
| gi_114703848_AC188397.3 | 13.66% | 96.65% | 7.23% | 90.64% | 23.78% | 99.85% | 24.99% | 98.74% |
| gi_112984829_AC187849.2 | 13.35% | 96.20% | 10.03% | 81.30% | 89.64% | 99.79% | 99.62% | 99.95% |
| gi_114329159_AC187206.2 | 12.79% | 90.98% | 17.15% | 86.94% | 94.92% | 99.91% | 100.00% | 100.00% |
| gi_124001821_AC197198.1 | 11.89% | 96.51% | 2.19% | 80.29% | 7.58% | 89.37% | 49.32% | 99.17% |
| gi_110625275_AC187066.2 | 11.43% | 93.54% | 9.90% | 85.10% | 89.64% | 99.43% | 98.38% | 99.76% |
| gi_145864560_AC202832.1 | 11.11% | 90.38% | 13.96% | 87.26% | 80.64% | 99.87% | 85.49% | 100.00% |
| gi_145864550_AC202824.1 | 10.44% | 94.13% | 16.86% | 88.31% | 13.72% | 97.60% | 30.45% | 94.91% |
| gi_110625271_AC187145.2 | 9.85% | 90.01% | 10.42% | 83.32% | 86.69% | 99.67% | 55.35% | 99.45% |
| gi_124001809_AC197186.1 | 8.96% | 88.67% | 3.89% | 82.47% | 7.85% | 87.67% | 35.52% | 99.61% |
| gi_114703850_AC190662.2 | 8.68% | 90.93% | 2.16% | 81.36% | 7.63% | 87.89% | 31.31% | 94.83% |
| gi_118601212_AC193955.1 | 8.34% | 92.46% | 1.92% | 80.29% | 11.84% | 89.19% | 29.41% | 96.45% |
| gi_111548773_AC189931.1 | 8.14% | 88.36% | 12.29% | 85.11% | 8.71% | 99.47% | 18.10% | 99.60% |
| gi_117960443_AC193480.1 | 7.45% | 91.55% | 12.57% | 81.59% | 21.12% | 98.44% | 32.39% | 99.04% |
| gi_108860751_AC187405.1 | 6.66% | 93.12% | 6.69% | 88.98% | 26.75% | 99.81% | 58.88% | 99.37% |
| Average | **43.87%** | 96.33% | **25.47%** | 88.86% | **62.48%** | 98.58% | **69.22%** | 98.62% |

***Supplementary Table 3.*** ***Comparisons the assembled scaffolds with BAC sequences of G. barbadense.***

| Query Name | Query Length (bp) | Target Scaffold | Scaffold Length (bp) | Coverage(Identity) of BAC region (%) |
| --- | --- | --- | --- | --- |
| BAC01 | 110,461 | scaffold3920 | 129,659 | 75.30(99.24) |
| BAC02 | 55,906 | scaffold4677 | 74,760 | 98.85(99.06) |
| BAC03 | 44,369 | scaffold11995 | 58,916 | 99.99(99.91) |
| BAC04 | 61,576 | scaffold4832 | 79,465 | 100.00(99.38) |
| BAC05 | 58,186 | scaffold7641 | 64,288 | 100.00(100.00) |
| BAC06 | 101,156 | scaffold8269 | 70,363 | 53.66(97.75) |
| BAC07 | 44,156 | scaffold11723 | 83,815 | 99.49(99.23) |
| BAC08 | 104,263 | scaffold1507 | 102,398 | 64.34(99.28) |
| BAC09 | 73,685 | scaffold4294 | 63,597 | 81.60(99.52) |
| BAC10 | 67,736 | scaffold11904 | 74,499 | 95.53(99.96) |

***Supplementary Table 4. Assessment of transcriptome sequence coverage of the G. barbadense genome assembly.***

|  | **≥ 90% identity and ≥ 50% length** | **≥ 90% identity and ≥ 90% length** |
| --- | --- | --- |
| ESTs | 16,804 (93.91%) | 15,116 (84.48%) |
| 454 | 1,893,631 (96.66%) | 1,656,035 (84.53%) |

***Supplementary Table 5. Summary of shotgun sequencing reads of other cottons mapped to assembled scaffolds of G. barbadense genome.***

| **Species** | **Lines** | **SRA accession number** | **Read length** | **Total reads** | **Base quality filter (Q >= 20)** | **Properly mapped reads** | **Mapping quality filter and remove PCR duplication** | **Base-pair coverage^*^** |
| --- | --- | --- | --- | --- | --- | --- | --- | --- |
| *G. herbaceum* | A1-155 | SRR847953 | 2 × 100 bp | 385,549,392 | 342,213,753 | 301,394,517 | 140,959,455 | 17.62 |
|  | A1-73 | SRR849166, SRR849173 | 2 × 100 bp | 202,723,343 | 197,154,604 | 174,524,018 | 120,210,660 | 15.03 |
|  | A1-97 | SRR617255, SRR617256, SRR617284 | 2 × 100 bp | 328,643,808 | 317,131,538 | 289,155,710 | 181,112,154 | 22.64 |
| *G. arboreum* | A2-1011 | SRR847923 | 2 × 100 bp | 412,359,904 | 368,888,845 | 327,718,246 | 185,653,133 | 23.21 |
|  | A2-34 | SRR847922 | 2 × 100 bp | 367,844,399 | 354,820,321 | 313,980,805 | 202,495,467 | 25.31 |
|  | A2-4 | SRR847945 | 2 × 100 bp | 343,470,023 | 340,453,145 | 297,584,726 | 181,155,673 | 22.64 |
| *G. raimondii* | D5-2 | SRR847961 | 2 × 100 bp | 152,913,856 | 151,304,541 | 139715777 | 74,422,478 | 18.61 |
|  | D5-31 | SRR847980 | 2 × 100 bp | 217,334,954 | 211,470,880 | 197,037,720 | 139,504,921 | 34.88 |
|  | D5-4 | SRR847974 | 2 × 100 bp | 109,951,143 | 109,147,477 | 105,256,556 | 65,656,420 | 16.41 |
|  | D5-53 | SRR847981 | 2 × 100 bp | 217,334,954 | 186,618,895 | 173,710,210 | 114,676,284 | 28.67 |
| *G. hirsutum* | Acala-Maxxa | SRR617482 | 2 × 100 bp | 452,976,869 | 447,280,941 | 406,093,573 | 291,849,420 | 23.35 |

^*^ Coverage was calculated based on the estimated A-genome size: 1.6 Gb; D-genome size: 800 Mb

***Supplementary Table 6. Assembled scaffolds assigned in subgenomes and anchored on the 26 pseudochromosomes of G. barbadense.***

|  | At subgenome | Dt subgenome |
| --- | --- | --- |
| Total scaffolds | 14,319 | 6,967 |
| Total length | 1,493,527,758 | 852,978,666 |
| Total genes | 36,946 | 34,575 |
|  |  |  |
| Anchored and oriented scaffolds | 7,841 | 4,476 |
| length of Anchored and oriented scaffolds | 1,195,000,455 | 802,309,298 |
| total genes | 32,530 | 32,321 |

**Supplementary Table 7. Chromosomes distribution of scaffolds.**

| Chromosome | Ordered | |
| --- | --- | --- |
|  | Scaffold Number | Scaffold Length (bp) |
| A01 | 533 | 83,454,191 |
| A02 | 550 | 89,731,153 |
| A03 | 691 | 102,275,589 |
| A04 | 427 | 66,299,034 |
| A05 | 614 | 93,810,923 |
| A06 | 800 | 100,034,992 |
| A07 | 544 | 87,365,090 |
| A08 | 683 | 108,674,945 |
| A09 | 517 | 79,449,896 |
| A10 | 681 | 107,075,239 |
| A11 | 598 | 96,770,036 |
| A12 | 648 | 93,480,475 |
| A13 | 555 | 86,578,892 |
| D01 | 329 | 62,968,913 |
| D02 | 472 | 71,317,061 |
| D03 | 283 | 48,575,973 |
| D04 | 279 | 51,938,909 |
| D05 | 383 | 60,343,712 |
| D06 | 341 | 66,700,274 |
| D07 | 378 | 61,301,369 |
| D08 | 352 | 69,128,419 |
| D09 | 282 | 53,850,888 |
| D10 | 340 | 64,637,854 |
| D11 | 340 | 65,624,333 |
| D12 | 361 | 63,542,810 |
| D13 | 336 | 62,378,783 |
| Total | 12,317 | 1,997,309,753 |

***Supplementary Table 8. Summary of gene models.***

| **Number of gene models** |  | **80,876** |  |
| --- | --- | --- | --- |
| Total length of gene model |  | 263.28 Mb |  |
| Gene models share in genome |  | 10.26% |  |
| Mean transcript length |  | 3,223.57 bp |  |
| Mean coding sequence length |  | 1,164.14 bp |  |
| Mean number of exons per gene |  | 5.16 |  |
| Mean exon length |  | 283.40 bp |  |
| Mean intron length |  | 423.41 bp |  |
| Mean 5'-UTR length |  | 180.80 bp |  |
| Mean 3'-UTR length |  | 318.33 bp |  |

***Supplementary Table 9. Summary of RNA-Seq libraries.***

| **Sample ID** | **Sample description** | **Read length** | **Reads passed quality filter** | **Reads mapped on genome** |
| --- | --- | --- | --- | --- |
| NE | Non-embryonic callus | 2 × 50 bp | 43,627,455 | 37,947,971 (86.98%) |
| RT | Root | 2 × 50 bp | 56,293,947 | 49,636,265 (88.17%) |
| SL | Stem cotyledon and true leaf mixture | 2 × 50 bp | 49,611,213 | 42,076,252 (84.81%) |
| SP | Stigma and petal mixture | 2 × 50 bp | 48,337,165 | 41,631,746 (86.13%) |
| AN | Anther | 2 × 50 bp | 49,568,343 | 42,675,772 (86.09%) |
| OV | -3, 0, 3 and 5-DPA Ovule mixture | 2 × 50 bp | 49,734,228 | 37,525,129 (75.45%) |
| FE | Fibre elongation  (7 and 12-DPA fibre mixture) | 2 × 50 bp | 50,679,039 | 46,168,172 (91.10%) |
| FS | Fibre second cell wall synthesis  (20 and 25-DPA fibre mixture) | 2 × 50 bp | 56,733,168 | 47,421,778 (83.59%) |

**Supplementary Table 10. Summary of annotations of *G. barbadense* gene models.**

| **Database/Source** | **Numbers** | **Percentage (%)** | **Comment** |
| --- | --- | --- | --- |
| Total | 80,876 | 100.0 | All proteins |
| Annotated | 77,297 | 95.6 | At least one annotation |
| Arabidopsis | 65,835 | 81.4 | Identity: ≥25%; length≥100aa; E-value≤1e-05 |
| Swissprot | 52,701 | 65.2 | Identity: ≥25%; length≥100aa; E-value≤1e-05 |
| TrEMBL | 72,885 | 90.1 | Identity: ≥25%; length≥100aa; E-value≤1e-05 |
| InterPro | 62,232 | 76.9 | At least one database; E-value≤1e-05 |
| Pfam | 60,501 | 74.8 | At least one Pfam domain; E-value≤1e-05 |
| KEGG | 23,015 | 28.5 | Has one KEGG term |
| GO | 48,960 | 60.5 | At least one GO term |

**Supplementary Table 11. The TE classification of whole genome, At-subgenome and Dt-subgenome for *G. barbadense.***

|  | *G. barbadense* | At-subgenome | Dt-Subgenome | Ungroup |
| --- | --- | --- | --- | --- |
| DNA | 289,691,263 | 159,345,498 | 106,405,116 | 23,940,649 |
| LTR | 58,414,688 | 27,894,997 | 25,414,833 | 5,104,858 |
| Gypsy | 874,707,632 | 599,577,430 | 214,222,256 | 60,907,946 |
| Copia | 219,089,277 | 114,919,613 | 83,570,252 | 20,599,412 |
| LINE | 97,601,619 | 50,700,750 | 38,850,438 | 8,050,431 |
| SINE | 3,203,526 | 1,804,885 | 1,152,718 | 245,923 |
| Retro | 86,679,801 | 57,733,024 | 23,019,229 | 5,927,548 |
| Others | 52,164,684 | 30,120,217 | 15,969,170 | 6,075,297 |
| Unclassfid | 97,069,771 | 55,893,689 | 32,967,309 | 8,208,773 |
| Total | 1,778,622,261 | 1,097,990,103 | 541,571,321 | 139,060,837 |

***Supplementary Table 12. Number and classification for every cluster.***

| Part_ID | Number | At | Dt | UN | Family | Clade | SubClade |
| --- | --- | --- | --- | --- | --- | --- | --- |
| Part1 | 133 | 56 | 66 | 11 | Copia | Tork | Tnt1 |
| Part2 | 107 | 47 | 44 | 16 | Copia | Tork | V12 |
| Part3 | 104 | 89 | 13 | 2 | Gypsy | Athila/Tat | Athila |
| Part4 | 166 | 52 | 99 | 15 | Gypsy | Athila/Tat | Tat |
| Part5 | 105 | 49 | 42 | 14 | Copia | Oryco |  |
| Part6 | 269 | 217 | 40 | 12 | Gypsy | Del | Peabody |
| Part7 | 123 | 92 | 27 | 4 | Gypsy | Del | Retrosat2 |
| Part8 | 26 | 16 | 8 | 2 | Copia | Retrofit |  |
| Part9 | 24 | 12 | 7 | 5 | Copia | Tork | Tto1 |

***Supplementary Table 13. Comparisons of full, solo and truncated LTR retrotransposons in the subgenomes of G. barbadense.***

|  | **Tetraploid** | | **Diploid** |
| --- | --- | --- | --- |
|  | **At-subgenome** | **Dt-subgenome** | **D5 genome** |
| Full LTR | 3,330 | 2,555 | 2,513 |
| Solo LTR | 8,626 | 3,718 | 5,001 |
| Truncated LTR | 10,864 | 4,321 | 5,451 |
| Solo/Full LTRs ratio | 2.59 | 1.46 | 1.99 |
| Truncated/Full LTRs ratio | 3.26 | 1.69 | 2.17 |

***Supplementary Table 14. Summary of SNPs detection.***

| **Genome** | **At-subgenome (P-value)** | **Dt-subgenome (P-value)** |
| --- | --- | --- |
| *G.* *herbaceum* (A1 genome) | 7,259,413 (1.0E-04) |  |
| *G.* *arboreum* (A2 genome) | 8,155,618 (1.0E-01) |  |
| *G.* *raimondii* (D5 genome) |  | 6,930,654 (0.03) |
| *G.* *hirsutum* (AD1 genome) | 4,579,965 | 2,924,914 |

***** Pearson's Chi-squared test with simulated p-value (based on 10000 replicates).

***Supplementary Table 15. Lineage specific SNPs in A genome phylogenies.***

| **SNP categories** | **A1 genome specific** | **A2 genome specific** | ***G. barbadense***  **At-specific** | ***G.* *hirsutum* At-specific** | **Specific between tetraploid and diploid** |
| --- | --- | --- | --- | --- | --- |
| Intergenic regions | 2,497,753 | 3,130,156 | 1,209,893 | 2,035,391 | 1,865,810 |
| Upstream of genes | 263,037 | 382,340 | 148,585 | 224,407 | 285,508 |
| Downstream of genes | 175,214 | 244,063 | 96,644 | 145,927 | 176,304 |
| Non-synonymous | 17,189 | 23,394 | 10,649 | 19,169 | 16,945 |
| Synonymous | 9,562 | 12,986 | 5,510 | 9,258 | 9,452 |
| Intron | 56,761 | 79,517 | 34,058 | 50,679 | 64,597 |
| **Total** | 3,019,516 | 3,872,456 | 1,505,339 | 2,484,831 | 2,418,616 |

**Supplementary Table 16.** Lineage-specific SNPs in D genome phylogenies

| **SNP Categories** | ***G. barbadense***  **Dt-specific** | ***G.* *hirsutum***  **Dt-specific** | **Specific between tetraploid and diploid** |
| --- | --- | --- | --- |
| Intergenic regions | 685,363 | 1,387,250 | 4,181,689 |
| Upstream of genes | 151,199 | 272,707 | 895,244 |
| Downstream of genes | 92,527 | 168,930 | 560,022 |
| Non-synonymous | 9,417 | 19,754 | 47,256 |
| Synonymous | 5,317 | 10,012 | 30,545 |
| Intron | 35,146 | 59,094 | 205,129 |
| **Total** | 978,969 | 1,917,747 | 5,919,885 |

***Supplementary Table 17. Categorization of biased and balanced expression of homoeologous genes in eight tissues.***

| **Tissues** | **Pairs of homeologous genes^*^** | **At biased** | **Dt biased** | **Balanced expression** |
| --- | --- | --- | --- | --- |
|  |  | **(log2(FC) >= 1)** | **(log2(FC) <= -1)** | **(-1 < log2(FC) < 1)** |
| NE | 1567 | 348 | 319 | 900 |
| RT | 883 | 196 | 169 | 518 |
| SL | 518 | 60 | 106 | 352 |
| SP | 766 | 207 | 211 | 348 |
| An | 624 | 168 | 147 | 309 |
| OV | 412 | 64 | 74 | 274 |
| FE | 708 | 212 | 187 | 309 |
| FS | 425 | 146 | 139 | 140 |
| **Total** | **5903** | **1401** | **1352** | **3150** |

^*^ Tissue-specific genes based on *k*-means clustering as shown in **Figure 4d**

**Supplementary Table 18. FPKM values of CesA genes detected by RNA-Seq.**

| **Gene IDs** | **Subgenome** | **Homologous in *Arabidopsis*** | **Clades** | **NE** | **RT** | **SL** | **SP** | **AN** | **OV** | **FE** | **FS** |
| --- | --- | --- | --- | --- | --- | --- | --- | --- | --- | --- | --- |
| Gbscaffold3390.13.0 | At | AtCESA8 | S1 | 0.03 | 5.28 | 23.66 | 0.61 | 0.08 | 0.15 | 0.38 | 522.92 |
| Gbscaffold16562.2.0 | At | AtCESA7 | S3 | 0.05 | 6.30 | 39.60 | 0.74 | 0.01 | 0.17 | 0.24 | 431.82 |
| Gbscaffold690.26.0 | At | AtCESA3 | S2 | 0.57 | 9.99 | 41.26 | 0.94 | 0.31 | 0.65 | 1.04 | 298.69 |
| Gbscaffold22349.4.0 | Dt | AtCESA8 | S1 | 0.07 | 3.49 | 19.84 | 0.77 | 0.07 | 0.13 | 0.53 | 186.27 |
| Gbscaffold3076.1.0 | Dt | AtCESA7 | S3 | 0.02 | 5.11 | 14.81 | 0.83 | 0.15 | 0.04 | 0.06 | 150.75 |
| Gbscaffold987.28.0 | Dt | AtCESA4 | S2 | 0.40 | 4.71 | 28.64 | 0.78 | 0.38 | 0.06 | 1.80 | 85.35 |
| Gbscaffold5097.28.0 | At | AtCESA10 | S3 | 0.03 | 3.26 | 12.57 | 1.68 | 0.07 | 0.02 | 0.01 | 54.23 |
| Gbscaffold13267.2 | Dt | AtCESA4 | S2 | 0.00 | 1.31 | 2.92 | 2.28 | 0.42 | 0.01 | 0.02 | 45.66 |
| Gbscaffold5573.1.0 | Dt | AtCESA3 | P2 | 54.74 | 117.37 | 88.80 | 117.70 | 11.91 | 84.57 | 161.30 | 37.92 |
| Gbscaffold5097.29.0 | At | AtCESA7 | S3 | 0.13 | 2.60 | 7.62 | 0.90 | 0.04 | 0.06 | 0.19 | 27.86 |
| Gbscaffold5097.31.0 | At | AtCESA1 | P1 | 56.26 | 87.08 | 82.43 | 91.87 | 14.29 | 40.92 | 115.33 | 26.22 |
| Gbscaffold35720.3.0 | Dt | AtCESA1 | P1 | 20.55 | 41.16 | 64.32 | 26.91 | 4.85 | 15.60 | 72.42 | 16.87 |
| Gbscaffold8073.1.0 | At | AtCESA2 | P3 | 15.66 | 11.58 | 38.08 | 15.30 | 0.78 | 5.47 | 19.68 | 14.86 |
| Gbscaffold13939.2.0 | Dt | AtCESA6 | P3 | 15.60 | 39.87 | 39.14 | 12.72 | 0.21 | 13.93 | 6.96 | 10.89 |
| Gbscaffold17798.2.0 | Dt | AtCESA3 | P2 | 1.60 | 5.00 | 5.78 | 2.00 | 3.43 | 2.74 | 5.42 | 10.53 |
| Gbscaffold1819.11.0 | At | AtCESA4 | S2 | 0.00 | 1.28 | 2.71 | 16.06 | 2.10 | 0.02 | 0.00 | 10.49 |
| Gbscaffold987.29.0 | Dt | AtCESA3 | S2 | 0.48 | 7.01 | 24.59 | 0.74 | 0.24 | 0.44 | 1.65 | 8.99 |
| Gbscaffold11266.16.0 | Dt | AtCESA3 | P2 | 12.96 | 6.75 | 15.11 | 5.03 | 4.24 | 8.22 | 15.45 | 6.37 |
| Gbscaffold5097.18.0 | At | AtCESA1 | P1 | 28.36 | 70.47 | 52.18 | 18.51 | 3.23 | 16.29 | 54.31 | 5.04 |
| Gbscaffold6974.1.0 | At | AtCESA6 | P3 | 6.75 | 35.18 | 12.36 | 7.46 | 1.19 | 4.72 | 13.38 | 4.57 |
| Gbscaffold24041.2.0 | At | AtCESA6 | P3 | 12.04 | 27.87 | 27.56 | 9.86 | 0.17 | 7.07 | 5.91 | 3.20 |
| Gbscaffold6316.16.0 | Dt | AtCESA6 | P3 | 7.22 | 7.81 | 24.20 | 12.34 | 1.46 | 5.52 | 7.75 | 3.02 |
| Gbscaffold7722.6.0 | At | AtCESA3 | P2 | 6.09 | 3.59 | 6.48 | 1.93 | 0.98 | 2.71 | 4.42 | 2.05 |
| Gbscaffold22349.3.0 | Dt | AtCESA8 | S1 | 0.03 | 4.12 | 13.00 | 0.40 | 0.02 | 0.08 | 0.57 | 0.99 |
| Gbscaffold4224.4.0 | Dt | AtCESA2 | P3 | 6.51 | 5.42 | 16.21 | 9.07 | 0.64 | 1.93 | 3.58 | 0.77 |
| Gbscaffold17720.1.0 | At | AtCESA2 | P3 | 8.26 | 10.00 | 22.64 | 11.89 | 0.80 | 4.31 | 3.48 | 0.57 |
| Gbscaffold5889.2.0 | At | AtCESA3 | P2 | 0.74 | 0.97 | 3.26 | 0.57 | 0.18 | 1.00 | 0.51 | 0.56 |
| Gbscaffold16350.11.0 | Dt | AtCESA8 | S1 | 0.02 | 0.45 | 0.95 | 0.11 | 0.00 | 0.20 | 0.13 | 0.36 |
| Gbscaffold3519.5.0 | Dt | AtCESA9 | P3 | 6.92 | 36.59 | 11.64 | 6.56 | 0.50 | 3.55 | 7.44 | 0.25 |
| Gbscaffold864.2.0 | Dt | AtCESA9 | P3 | 1.10 | 0.62 | 1.44 | 0.61 | 0.11 | 0.59 | 0.44 | 0.03 |
| Gbscaffold13186.7.0 | At | AtCESA9 | S1 | 0.00 | 0.94 | 0.24 | 0.06 | 0.03 | 0.02 | 0.07 | 0.02 |
| Gbscaffold864.3.0 | Dt | AtCESA6 | P3 | 0.93 | 0.47 | 0.64 | 0.31 | 0.05 | 0.65 | 0.36 | 0.00 |
| Gbscaffold29439.3.0 | At | AtCESA2 | P3 | 0.84 | 0.49 | 1.13 | 0.68 | 0.10 | 0.16 | 0.26 | 0.00 |
| Gbscaffold29439.2.0 | At | AtCESA6 | P3 | 0.73 | 0.25 | 0.93 | 0.41 | 0.09 | 0.17 | 0.11 | 0.00 |
| Gbscaffold29439.1.0 | At | AtCESA6 | P3 | 0.33 | 0.16 | 0.29 | 0.21 | 0.08 | 0.15 | 0.08 | 0.00 |
| Gbscaffold8728.8.0 | Dt | AtCESA9 | P3 | 0.00 | 0.00 | 0.03 | 0.00 | 0.00 | 0.00 | 0.00 | 0.00 |
| Gbscaffold15929.4.0 | At | AtCESA9 | P3 | 0.00 | 0.00 | 0.01 | 0.00 | 0.00 | 0.00 | 0.00 | 0.00 |

***Supplementary Table 19. Gene-specific PCR primers developed for qRT-PCR validation of CesA genes.***

| Gene IDs | qRT-PCR primer | |
| --- | --- | --- |
|  | Forwad (5' to 3') | Reverse (5' to 3') |
| Gbscaffold3390.13.0 | GCAGTGTGTCTACATTGGAT | CATTTGTTGCTCAGGTGGGATT |
| Gbscaffold22349.4.0 | GCAAGTAAGTGGACTGAAACAT | CATACATAATCAGCATCAAA |
| Gbscaffold690.26.0 | TTTGCCACTACTGTTGATTTG | TTGAACCAACAAAATCATAGG |
| Gbscaffold987.28.0 | TATTTTTCCTTTTTGCCACTA | AATCACACATTTTGCTCTTGC |
| Gbscaffold16562.2.0 | AGTGTTCCCGTTCCTTCTTTG | CATCTCCATTTTGCTTGTTCATAC |
| Gbscaffold3076.1.0 | ATTCCCTCCTTTCATATCCT | CGACCGTCAACCCAATTTC |
| Gbscaffold5097.31.0 | CTTTGTGTTCACTTCTATTCGTC | AAGGCTTCCTCTTCTTTTTATTT |
| Gbscaffold5097.18.0 | ATGAAAGGGACTTGTTACTGT | CCCAAACTTATTCCAATCTGA |
| Gbscaffold35720.3.0 | ATAACCAAAGGAAAAAGAGAC | TTACACAACACCAACATCAAA |
| Gbscaffold7722.6.0 | ACTGTCACTTTCGTTTCTTA | ACGTGCTTCTTTCTTTAGCT |
| Gbscaffold5573.1.0 | AGAACCTATAAAGACTGAAG | CAAAGTAGAAACAAGATGAC |
| Gbscaffold6974.1.0 | AATTATCCTACTCCGGCTTGTGGTC | CGGTCAAGGTATGTTTCTCGTTCAA |
| Gbscaffold3519.5.0 | CTGATCCTTCCTTGACTTTGCCA | CAATCCTCCATTCTTTCCTTCCA |

***Supplementary Table 20. PCR primers of mixed scaffold and comparisons the sequencing result of PCR product with the scaffolds.***

| Scaffold ID | Foward Primer | | | Reverse Primer | | | PCR product length | Similarity |
| --- | --- | --- | --- | --- | --- | --- | --- | --- |
|  | Start site | End site | Primer sequence | Start site | End site | Primer sequence |  |  |
| scaffold2632 | 484551 | 484570 | TTCGTTGGGTCATTGTTAGC | 484936 | 484955 | GCATTTGGTGTAGGTTTGGA | 405 | 100 |
| scaffold739 | 236360 | 236378 | GTGTCTCTGCCTGTGTGGT | 236959 | 236977 | TTACGGATCTTGATTTGGA | 618 | 95 |
| scaffold4634 | 372153 | 372172 | AAACTTCAGGTGAGTCTTCG | 372708 | 372689 | ACAATTTCTTGCTTTGCTAC | 556 | 100 |
| scaffold13980 | 209463 | 209482 | GCTCGTCTTGATGTTATTCG | 209898 | 209879 | ACTTAAGGCAGTTTTGGTTG | 436 | 98 |
| scaffold5968 | 182079 | 182098 | GGACTCGTTTTCACCTTCTC | 182736 | 182717 | AAATTTTCAGCCCGTTTACT | 658 | 89 |
| scaffold7290 | 239394 | 239413 | TTTCGTTTCAGTATGCCTCT | 240163 | 240144 | TTTCTTTACCCCGATTGTTC | 770 | 100 |
| scaffold10295 | 159198 | 159216 | TTTGGGGAGTTGAAGGAAG | 159812 | 159794 | AATAGGGTCGGAAGCGTAT | 615 | 88 |
| scaffold7492 | 349449 | 349467 | ACTTCTGCCCCCATACACC | 350151 | 350133 | AATAATCCCGTCCCTCCAA | 703 | 100 |
| scaffold4149 | 71406 | 71427 | TGAACTAAGAATAAGGAGGCAG | 72019 | 71998 | TTGGAAGTCAAAACGAATAAAG | 614 | 100 |
| scaffold2694 | 76992 | 77010 | AACCATTTTAGCCCCGAGA | 77526 | 77508 | AAGCCCAAGTTTGAACCCA | 535 | 100 |
| scaffold4758 | 137792 | 137811 | GGCTCTTTGGACTTGGTGGT | 138509 | 138490 | GGAGGCATTTAATCTGGACG | 718 | 100 |
| scaffold987 | 423533 | 423552 | AGGTGAAGGTGATTGTGTGC | 424098 | 424079 | AAGCGTGGATCTGTTCTGTG | 566 | 100 |
| scaffold2289 | 421696 | 421715 | CCTGAAAAGAGGTGGGGCTA | 422391 | 422372 | TTTGAATCAAGTTGGGAGCG | 696 | 100 |
| scaffold2575 | 170947 | 170966 | AAGCTCCCCAGTTACTATCA | 171598 | 171617 | TTTCTTTTCCATTTCTCTCA | 704 | 86 |
| scaffold2793 | 336192 | 336211 | TGTTTGATTCGTTTTGTTCG | 336832 | 336851 | ATGTTGATTTCTATTGCGGC | 660 | 100 |
| scaffold5018 | 329213 | 329232 | ACATCACCTTTCACCTTCCG | 329613 | 329632 | TCACCCCCTTCCTCTACTCC | 445 | 99 |
| scaffold7851 | 143839 | 143858 | TACCCGTTTTCTCATTTTGG | 144491 | 144510 | CGCTGTGTCCTTTTTACTTT | 676 | 96 |
| scaffold1393 | 182826 | 182845 | AAACCCAAAACCAAATGAGC | 183466 | 183485 | TATGAATTGACGAACCCGAA | 696 | 86 |
| scaffold10824 | 408464 | 408483 | CTCGCCTCACTCAAAAAAAT | 409014 | 409033 | ACAAAAAGCTACCCAACAAA | 570 | 84 |
| scaffold2715 | 346560 | 346581 | CATCAATATGTTTTGGTGGTTT | 347306 | 347327 | AGAGAAAGGAGAGGAGAAAGCT | 768 | 100 |

**Supplementary Table 21. The CesA gene family of *Arabidopsis* (AT), *G*. *raimondii*, *G*. *arboreum*, *G*. *hirsutum* and *G*. *barbadense.***

| Clade | List of AT | AT | Gr (Paterson et al.) | Ga (Li et al., Ananlysis by us) | TM-1(Li et al.) | | | TM-1(Zhang et al.) | | | Gb(this study) | | |
| --- | --- | --- | --- | --- | --- | --- | --- | --- | --- | --- | --- | --- | --- |
|  |  |  |  |  | At | Dt | At+Dt | At | Dt | At+Dt | At | Dt | At+Dt |
| P1 | At1,10 | 2 | 2 | 1 | 0 | 2 | **2** | 2 | 2 | **4** | 2 | 1 | **3** |
| P2 | At3 | 1 | 3 | 3 | 4 | 3 | **7** | 3 | 3 | **6** | 2 | 3 | **5** |
| P3 | At2,5,6,9 | 4 | 4 | 6 | 6 | 5 | **11** | 5 | 6 | **11** | 8 | 7 | **15** |
| S2 | At4 | 1 | 2 | 2 | 3 | 2 | **5** | 2 | 2 | **4** | 2 | 3 | **5** |
| S3 | At7 | 1 | 2 | 2 | 1 | 2 | **3** | 2 | 2 | **4** | 3 | 1 | **4** |
| S1 | At8 | 1 | 2 | 2 | 2 | 2 | **4** | 1 | 2 | **3** | 2 | 3 | **5** |
| Total | | 10 | 15 | 16 | 16 | 16 | **32** | 15 | 17 | **32** | 19 | 18 | **37** |

# Supplementary Notes

## 1. Sequencing and scaffold assembly of the *G. barbadense* genome

***Plant material and genomic DNA preparation*** *G. barbadense* acc. 3-79 (a doubled-haploid from Pima germplasm) was obtained from the Cotton Research Institute (CRI) of the Chinese Academy of Agricultural Sciences (CAAS) in 2002 and self-fertilized to maintain homozygosity in each generation. Fresh young leaves were collected, immediately frozen in liquid nitrogen and stored at -80 °C until DNA extraction. Genomic DNA was extracted using the standard CTAB method^1^.

***Library construction, sequencing and quality control*** Genomic DNA was sheared into fragment sizes of ~300 bp for shotgun library construction following the standard Illumina protocols (www.illumina.com), and large DNA fragments (size range 5, 10 and 20 kb) were isolated for DNA-PET library construction following our previously published protocols with minor modifications^2,3^. Libraries were sequenced using the Illumina Genome Analyzer II platform. In total, 470.93 Giga base pairs (Gb) of sequence data were generated. The raw reads were processed to remove those of low quality as well as adaptor sequences and possible contaminated reads of bacterial and viral origin using Cutadapt^4^ and SSearch software^5^. After filtering, we obtained 446.58 Gb of high quality reads, representing 173-fold base-pair coverage and 5,846-fold physical coverage of the *G. barbadense* genome (**Supplementary Table 1**).

***Genome size estimation*** The genome size was estimated based on the k-mer depth distribution of shotgun reads, as described by Li *et al*. and Ling *et al*.^6,7^, a method which has been used to accurately estimate the genome sizes of a number of organisms based on short tag sequences. The Jellyfish program^8^ was used to count the number of occurrences of every k-mer. We tested multiple k-mer sizes, ranging from 13 to 31, to count k-mer frequency. We observed that different size k-mers shared similarly shaped frequency distributions (**Supplementary Figure 1**). For the 31-mer, the k-mer depth distribution peaked at 48 (**Supplementary Figure 1**). Using the following empirical formula derived from the original [ M = (N/G)×(L-K+1)/L ]:

G=N×(L-K+1)/(L×M)

We calculated the genome size of *G. barbadense*, where N represents all sequencing base pairs (184,321,882,272), L is the average length of quality reads (91), K is the k-mer size used for estimation (31), M is the peak of 31-mer depth distribution (48). Thus,

G = 184,321,882,272 bp × (91-31+1) / (48 × 91) = 2,574,092,220 bp

yielding an estimated genome size of 2.57 Gb.

***Genomic scaffold assembly*** Before assembly, substitution sequencing errors in the shotgun and DNA-PET reads were corrected using Quake^9^. We used assembler SOAPdenovo2 for genome assembly. First, we used 63-mer from all sequencing reads (including shotgun and DNA-PET library reads, ignoring pair information) to construct contigs. Second, the pair-end reads from libraries with different fragment sizes were realigned to the contigs using 63-mer. Step by step, the pair-end read libraries with small to large insertion sizes were used to build scaffolds. Finally, unmapped reads were used to fill the gaps inside the scaffolds using GapCloser^10^. All sequences, including the paired end shotgun and DNA-PET reads assembled for each scaffold, were uploaded to Gap5^11^ for alignment and visual inspection. An example of scaffolds is provided in **Figure 3b**. To avoid the contamination of organelle genome sequences in the assembled scaffolds, we aligned the *G. barbadense* chloroplast and Arabidopsis thaliana mitochondrion sequences to scaffolds noting that only 250 scaffolds (0.8% of all scaffolds) were hit by chloroplast and that two were hit by mitochondrion sequences. Those scaffolds matching the organelle genome sequences were mostly short (approximate 2 Kb), and were removed before further analysis.

***Assessment of scaffold assembly quality*** We used the BAC clone and transcriptome sequences to evaluate the quality of the assembly. We have previously constructed a BAC library of *G. barbadense* (unpublished) including 153,600 clones with an estimated average insertion size of 110 kb for a 6.5-fold coverage of the genome. From this library, 10 BAC clones were randomly selected for sequencing analysis by Illumina GAII platforms. The BAC sequences of each clone were assembled using Velvet 1.1^12^, and further optimized using the VelvetOptimiser version2.2.5^13^ (The parameter is “-s 31 -e 127 -t 8 -k max”). The largest contigs in each BAC clone sequences were used to search the assembled scaffold pool using lastz (--gfextend --chain --gapped --identity=80..100 --matchcount=200 --format=general). Nine of ten BAC sequences were matched to single scaffolds with more than 90.5% coverage and 99.5% identity averagely, except BAC06 (**Supplementary Table 3; Supplementary Figure 3**).

To evaluate the coverage of the *G. barbadense* transcripts in the assembly, transcriptome sequences of 17,894 expressed sequence tags (ESTs) generated by Sanger sequencing and 1,959,060 reads generated by 454/Roche were mapped to the assembled *G. barbadense* genome scaffolds. More than 84% of the transcriptome sequences were covered by scaffolds with ≥ 90% identified and ≥ 90% covered (**Supplementary Table 4**).

## 2. Assignment of assembled scaffolds to subgenomes of *G. barbadense*

The available shotgun sequencing short read datasets of *G.* *herbaceum* (A1 genome, 3 lines), *G.* *arboreum* (A2 genome, 3 lines), *G.* *raimondii* (D5 genome, 4 lines) and *G.* *hirsutum* (AD1 genome, 1 line) were downloaded from the NCBI SRA database (**Supplementary Table 5**). The shotgun reads of each dataset (line) were mapped to the *G. barbadense* genome assembly using the BWA programme with default settings^14^ in order to characterize the subgenome origin of the assembled scaffolds. After mapping, the shotgun reads with low mapping scores (< 20) were filtered using SAMtools programme^15^. In addition, the redundant reads potentially from PCR amplification were removed using Picard program^16^. For each assembled scaffold, the percentages of the base-pairs covered by the shotgun reads from each dataset were calculated using BEDtools^17^. The following criteria were used to call if a *G. barbadense* scaffold belongs to either At or Dt: 1) more than 40% of a scaffold was covered by shotgun reads derived from diploid A1, A2 or D5 progenitor genomes; 2) the log_2_ ratio of average base-pair coverage between A1 or A2 to D5 diploid progenitor genome is more than 2; 3) the ratio difference is significant (*p* value < 0.01) as determined by Student’s t-test using the shotgun read datasets derived from multiple lines of each diploid genome (A1, A2, D5) as replicates. Scaffolds that did not satisfy with these three criteria were regarded as ungrouped.

Our mapping indicated that the shotgun sequencing reads from the diploid A1 and A2 genomes were highly correlated (R^2^ = 0.97) when aligned to *G. barbadense* genome scaffolds (**Supplementary Figure 4a**), consistent with the close relationship between A1 and A2 genome clades. In contrast, the *G. barbadense* scaffolds were distinguished by two groups, one covered by the shotgun reads from the diploid A genome clades (A1 and A2), and the other covered by the shotgun reads from the D genome clades (D5) (**Supplementary Figure 4b**). Using the criteria described above, we assigned approximately 1,494 Mega base pairs (Mb) to the At-subgenome and 853 Mb to the Dt-subgenome, respectively (**Supplementary Table 6; Supplementary Figure 4c**). The remaining ungrouped scaffolds were largely with short length (**Supplementary Figure 4d**).

In the ungrouped scaffolds, we identified 77 that contained hybrid sequences, one part from the At and the other part from the Dt (**Figure 3a**). In order to validate the authenticity, PCR primers were designed and the PCR product were spanned the hybrid point. Then, the PCR product were sequenced by Sanger ABI 3730xl (**Supplementary Table 20**). The sequence result was aligned with scaffold using the bl2seq program of BLAST soft package.

## 3. Genome annotation

***Analysis of repetitive sequences*** There are two main types of repeats in the genome: tandem and interspersed. Tandem repetitive sequences were identified using Tandem Repeats Finder (TRF)^20^. The interspersed transposable elements (TEs) were identified in DNA and in protein. For DNA we used six *ab* *initio* software packages, LTR_STRUC^21^, LTRharvest^22^, MGEScanLTR^23^, TransposonPSI^24^, RepeatModeler^25^ and MITE_Hunter^26^. The de novo TE library of *G. barbadense* was built by merging the repetitive sequences identified using the CDHIT package^27^. RepeatMasker was then used to search the *G. barbadense* genome against a combined library of Repbase^28^ (version 17.0.1) and the de novo TE library of *G. barbadense*. For proteins, RepeatProteinMask was used to search the *G. barbadense* genome against the Repbase. The TEs identified by both approaches were combined to gain a comprehensive view of TEs in the *G. barbadense* genome. In addition, all identified repeats were classified using TEclass^29^, Repclass^30^ and the HMM profiles from GyDB^31^.

***Prediction of protein-coding gene*** We used three approaches to predict protein coding genes: the de novo method, the homology-base method and the transcriptome-base method. The results were then integrated using EVM software^32^.

For *de novo* prediction, the repeat-masked *G. barbadense* genome was entered into AUGUSTUS^33^, GeneID^34^, GlimmnerHMM^35^, FGENESH^36^ and GenScan^37^ using parameters that were trained by the Arabidopsis genome.

In the homology-based method, the protein sequences from plants with known whole genome sequences , Arabidopsis^38^ (TAIR release 10), Papaya^39^, Grape^40^, Cacao^41^ and Populus^42^, were aligned to the *G. barbadense* genome using genBlastA^43^ with an e-value less than 1×10^-5^. To obtain accurate spliced alignments, the matching proteins were aligned to the homoeologous genome sequences using GeneWise^44^.

In the transcriptome-based method, 17,894 ESTs and 1,959,060 454/Roche transcriptome sequences were mapped to the *G. barbadense* genome to generate spliced alignments, which were linked using PASA after removing the overlaps^45^. The RNA-seq data sets were also mapped to the *G. barbadense* genome using the default settings of Tophat and the alignment data were subjected to Cufflinks (using default parameters) to identify spliced transcripts^46,47^.

Resultant data were combined by EVM to produce consensus gene models. Those that were supported by only one *de* *novo* gene prediction method and that also contained TE family domains were filtered. The remaining gene models were further processed using PASA^48^ to identify UTR regions and alternative splice events using transcriptome data. These gene models are summarized in **Supplementary Table 8**.

***Gene functional annotation*** The predicted protein-coding genes in *G. barbadense* were annotated based on alignment to the TAIR, SwissProt and TrEMBL databases by BLASTP with cutoff of e-value 1×10^-5^, identity ≥ 0.25 and alignment length ≥ 100 amino acid. InterPro was used to annotate motifs and domains through comparisons with publicly available databases including Pfam, PRINTS, PROSITE, ProDom and SMART. Putative gene pathways were derived from genes matched in the KEGG database. The Gene Ontology (GO) information for each gene code was extracted from InterPro. Gene model annotations are summarized in **Supplementary Table 10**.

## 4. Gene expression analysis by RNA-Seq

***Tissues***  To obtain a comprehensive view of the *G. barbadense* transcriptome and to assess the expression of each individual gene, we analyzed whole transcriptome by RNA-Seq in eight different cotton tissues or developmental stages, including NE(Non-embryonic callus), RT(Root), SL(Stem cotyledon and true leaf mixture), SP(Stigma and petal mixture), AN(Anther), OV(-3, 0, 3 and 5-DPA Ovule mixture), FE(Fibre elongation, 7 and 12-DPA fibre mixture) and FS (Fibre second cell wall synthesis, 20 and 25-DPA fibre mixture). The sample descriptions are shown in **Supplementary Table 8**. Tissues from root, stem, cotyledon and leaves were harvested in two true-leave stages from plants that were grown in a growth chamber with 16 hours of light and eight hours of darkness (28 ℃). Anthers, stigma and petal were collected during anthesis from plants cultivated in the field after being grown via normal farming practices. Ovule and fibre tissues were sampled from plants grown in normal field conditions. Ovule was collected at -3, 0, 3 and 5 days post anthesis with fibres (DPA), while fibre tissues were collected at 7 and 12 DPA (at FE) and at 20 and 25 DPA (at FS). All samples were quick frozen in liquid nitrogen and stored at -80℃ until use.

***RNA-Seq*** Total RNAs were isolated using a modified guanidine thiocyanate method^49^, and cDNA libraries were constructed and sequenced according to Illumina’s protocols (www.illumina.com). Low quality RNA-Seq reads were removed from raw data using the FASTX-toolkit^50^. For each library, approximately 50 million high-quality paired-end reads were obtained and mapped to *the G. barbadense* genome assembly using default parameters of TopHat^46^. For each library, more than 85% of paired-end reads were mapped onto the *G. barbadense* genome (**Supplementary Table 9**). For gene expression, the FFPKM (Fragments Per Kilobase of transcript per Million mapped reads) were calculated using Cufflinks^47^.

## 5. TE evolution in the subgenomes of *G. barbadense*

The LTR_STRUC programme was used to identify full length LTR retrotransposons. LTRs were then classified by aligning the HMM profiles to the GyDB2 database^31^ and according to the rules described by Wicker^51^. We aligned the 5- and 3-ends of the LTR sequences of each retrotransposon using MUSCLE^52^ and calculated the divergence (K) under the Kimura two parameter (K2P) model using the distmat program of EMBOSS toolkit^53^. The divergence time of LTR was estimated using the formula T = K/2r, where r represents a synonymous substitution rate of 1.3 × 10^-8^ per site per year. An all-by-all BLASTN (e-value < 1×10^-5^) was performed using the LTR sequence of 2,825 intact LTR retrotransposons. The LTR retrotransposons were clustered into putative families according to reciprocal blast similarity. Force-directed graph drawings were generated using Cytoscape^54^. The corresponding phylogenetic tree was drawn from each cluster using MEGA 6.0 with a neighbor-joining mode and 1000 bootstrap tests^55^. The solo and truncated LTRs were identified using methods previously described^56^.

We noted that the largest fraction of TEs in the allotetraploid genome was comprised of LTR, with the *Ty3*/*Gypsy* superfamily being more abundant than the *Ty1*/*Copia* superfamily (**Figure 2a; Supplementary Table 11**). The *Gypsy* and *Copia* superfamilies accounted for 214.2 Mb (25.1%) and 83.6 Mb (9.8%) of the Dt, respectively, consistent with their proportions in the *G.* *raimondii* genome. However, we observed that the *Gypsy* superfamily accounted for a high percentage of the At (599.6 Mb, 40.2%) relative to Dt (**Figure 2a; Supplementary Table 11**), suggesting that a substantial proliferation of *Gypsy* retrotransposons was responsible for the expansion of the diploid At or A-genome. Therefore, we used a sequence similarity clustering approach to represent the LTR retrotransposons in the At and Dt. The clustering patterns indicated that specific LTR retrotransposon clusters preferentially occurred in At, with the exception of several shared clusters (**Fig. 2b**). In order to trace the history of LTR retrotransposons in the genus *Gossypium*, we estimated the timing of insertion for LTR retrotransposons. The data indicated that a burst of LTR retrotransposon amplifications must have occurred very recently in Dt, peaking within the last few million years (1.9 Myr), whereas At appeared to have undergone a surge of retrotransposon amplification approximately 3.1 Myr ago (**Fig. 2c**). Just as TEs have been implicated in the growth of genome size, their amplification and genomic presence can also lead to contraction via unequal homologous recombination or non-homologous recombination, which are typically evidenced by solo LTR or truncated LTR retrotransposons^57^. We observed higher ratios of solo and truncated LTRs to intact LTR in At relative to Dt (**Supplementary Table 13**). While Dt and its diploid progenitors have similar ratios of solo and truncated LTRs to intact LTR in their genome/subgenome (**Supplementary Table 13)**. These results indicated that the At accommodated a lower level of LTR growth after proliferation. Thus, we conclude that proliferation of LTR retrotransposons played a dominant role in shaping the landscape of the At.

We noted that TEs exhibited distinct behaviors in the two diploid progenitors of the allotetraploid. However, the gene organization along the chromosomes was highly conserved in the diploid progenitors as demonstrated in **Figure 1**. These results indicated that the mechanisms of TE proliferation underlying the twofold difference in genome sizes operate heterogeneously in genomic regions, leaving some regions relatively unchanged while affecting others more dramatically. We observed that the conservation of gene collinearity was disrupted at the heterochromatic region along the scaffolds of At, demonstrating that genome expansion preferentially occurred in the existing heterochromatin region. However, it is known that variations in heterochromatin can create species barriers^58^. Thus, the unequal accumulation of TEs, particularly LTR retrotransposons, in heterochromatic regions of an ancestor species may have played an important role in the speciation of A-genome lineages.

Taken together, these analyses provide evidence, at the genomic scale, that LTR retrotransposons played an important role in shaping the genome during the evolution of the genus *Gossypium*.

## 6. SNP calling and lineage-specific SNP divergence

After filtering reads with low read scores (< 20) and accounting for PCR redundancy, the short sequencing data that were mapped to the assembled *G. barbadense* genome and those that were derived from the diploid progenitors (A1, A2, and D5) and from the tetraploid *G. hirsutum* (AD1) were subjected to SNP detection using GATK^59^. The mapping files that were derived from the same diploid genome clade were pooled together for SNP calling using the following parameters:

-stand_call_conf 50.0;

-stand_emit_conf 10.0

-dcov 500

-ploidy 4

To remove low quality SNPs, the following parameters were applied:

-clusterWindowSize 10

-filter "QD<2.0" -filterName QualByDepth

-filter "MQ<=40.0" -filterName MapQual

-filter "QUAL<100" -filterName QScore

-filter "MQ0>=10 && ((MQ0 / (1.0 * DP)) > 0.1)" -filterName MapQualRatio

-filter "FS>60.0" -filterName FisherStrandBias

We used SnpEff^60^ with default parameters to annotate SNPs, using *G. barbadense* as reference. SNPs were categorized according to gene position as intergenic, upstream (within 5 kb of upstream) or downstream (within 5 kb of downstream) and as introns or exons.

Shotgun sequencing of representatives from the diploid progenitor clades was used to gain a comprehensive overview of the frequency and distribution of genetic variation in the allotetraploid and diploid progenitors. In the allotetraploid genome, we observed that 0.49% (7.26 million SNPs) and 0.55% (8.16 million SNPs) of the At diverged from the A1 (*G.* *herbaceum*) and A2 (*G.* *arboreum*) diploid genome, respectively, while 0.81% (6.93 million SNPs) of the Dt diverged from the D5 (*G.* *raimondii*) diploid genome (**Supplementary Table 14**). In contrast, we noted that approximately 0.31% (4.58 million SNPs) and 0.34% (2.92 million SNPs) of At and Dt diverged from the two allotetraploid genomes *G. barbadense* and *G.* *hirsutum*, respectively (**Supplementary Table 14**).

To address why the phenotypes of allotetraploid cotton vary more than those of their diploid progenitors, it is essential to analyse the lineage-specific genetic variations in the subgenomes and in those of their diploid progenitors. In the phylogenetic context of the diploid A-genome and the tetraploid At, we identified 2.4 million SNPs that differed in the At of allotetraploids (*G. barbadense* and *G.* *hirsutum*) relative to the diploid A-genomes (A1, *G.* *herbaceum* and A2, *G.* *arboreum*), indicating that these genetic variations occurred after allotetraploidization (**Supplementary Table 15**). We identified 1.5 and 2.5 million lineage specific SNPs for the At of *G. barbadense* and *G.* *hirsutum*, respectively. The number of SNPs identified for the allotetraploid was slightly lower than the number identified for the A1 (3.0 million lineage specific SNPs) and A2 genomes (3.9 million lineage specific SNPs) (**Supplementary Table** **13**). Similarly, we identified 5.9 million SNPs that differed in the Dt of allotetraploids (*G. barbadense* and *G.* *hirsutum*) relative to the diploid D-genome (*G.* *raimondii*) (**Supplementary Table 16**) while we observed that the Dt of *G. barbadense* and *G.* *hirsutum* held only 1.0 million and 1.9 million lineage- specific SNPs, respectively (**Supplementary Table 16**).

SNPs represent the most abundant forms of genetic variation in the genome. To better understand the evolution of the allotetraploid genome, we conducted a comparative analysis of identified SNPs to track lineage-specific SNP divergences. According to the SNP annotations, the linage-specific SNPs were categorized into non-synonymous, synonymous and other (i.e., intergenic, upstream of gene, downstream of gene and intron) (**Supplementary Table 15 and Supplementary Table 16**).

## 7. Gene family cluster

We used the OrthoMCL^61^ software (version 2.0.3) to classify the complete set of protein-coding genes from the D5 genome (*G.* *raimondii*^62^) and the At and Dt of *G. barbadense* into narrowly defined gene lineages. Firstly, the all-vs-all blastp procedure was conducted using primary protein sequences in these three genome/subgenomes (E-value 1e-5). Then, the result of blastp was loaded into a local mysql database. Finally, we used the mcl algorithm to classify them into orthologous clusters (--abc -I 1.5). A customized Perl script was programmed to count gene numbers of each cluster. These orthologous clusters could represent the evolutionary history of gene families, including genes that diverged from a common ancestor and genes that expanded or contracted in one genome or subgenome. We identified 20,378 orthologous clusters in the subgenomes of *G. barbadense* and in the D5 genome. In the orthologous clusters, 10,012 clusters belonged to 1:1:1 orthologous relationships (**Supplementary Figure 7a**), indicating that these gene families descended from a single gene present in the last common ancestor. We observed that 5,283 orthologous clusters, including approximate 12,000 genes in each genome/subgenome, contained putative gene duplications at least in one genome or subgenome. Among these orthologous clusters, a substantial majority of the genes (about 6,500 genes in each genome/subgenome) have undergone multiple duplication events in all genome and subgenomes. We further analyzed the functions of these genes noting that they are involved in processes of cellular polysaccharide biosynthesis and cellulose biosynthesis, microtube-based movements and response to hormonal stimuli (**Supplementary Figure 7c**). In contrast, the genes presented in the 1:1:1 orthologous clusters were enriched for basic cellular functions (**Supplementary Figure 7b**). These results support the conclusion that the duplications of some functional genes might have been advantageous during speciation as the organism adapted to new environments^63,64^.

We applied HMMER (version 3.0)^65^ to identify the gene families of transcription factors (TFs) in the *G. barbadense* genome using the PlnTFDB^66^ (Plant Transcription Factor Database) to classify the TFs. We identified a total of 4,843 TF genes, which were categorized into 58 TF gene families. The top 5 TF gene families were bHLH, MYB, ERF, C2H2 and NAC. We further compared the TFs gene families in the *G. barbadense* and *G.* *raimondii* genomes noting that most of them showed a two-fold difference between the two with correlations as high as 0.71, consistent with known differences between tetraploid and diploid genomes. Some of the TF families, such as GRAS, E2F/DP and those related to MYB were expanded in the *G. barbadense* genome, while some (such as Dof, M-type and FAR1) were contracted in the *G. barbadense* genome relative to that of *G.* *raimondii* (**Supplementary Figure 8a**). In addition, we compared the TF families in the At and Dt of *G. barbadense* observing that three of them (MYB, WRKY and bZIP) were overrepresented in the Dt, while four (C2H2, NAC, B3 and the family related to MYB) were overrepresented in the At (**Supplementary Figure 8b**).

## 8. Identification of homoeologous genes

To identify homoeologous genes, we associated genes in At and Dt with those in the D5 reference genome using MCscanX with default settings^67^, allowing us to compare the order of genes in the tetraploid subgenomes to that of the diploid reference genome. After removing the tandem duplications and multiple matches, syntenic blocks containing more than five aligned protein gene pairs were identified; these were regarded as homoeologous gene pairs. We identified a total of 6,461 homoeologous gene pairs in the *G. barbadense* genome.

To understand the roles played by homoeologous genes in the transcriptome during cotton development, we investigated the expression profiles of these genes in eight different tissues. The RNA-Seq FPKM values were normalized for each of the homoeolgous genes using Genesis^68^ with expression values in the range of -3 to 3. The k-means clustering method was applied to identify and cluster highly expressed homoeologous genes in each tissue. A heatmap was used to illustrate these results.

## 9. Phylogenetic and expression analyses of genes potentially involved in fibre development

We performed detailed phylogenetic analyses of six gene families (β-tubulin, actin, profilin, FLA, APX and LIM). The hmmsearch component of the HMMER package and direct BLASTP were adapted to identify all family members in the diploid D5 genome, the tetraploid At- and Dt- subgenomes and the *Arabidopsis* genome. For every gene family, putative sequences were manually checked and aligned using ClustalW^70^. Phylogenetic analysis was conducted in MEGA6.0 with 500 bootstrap replications. We also investigated the expression profiles of the six gene families. These results are presented in **Supplementary Figure 9**.

***Actin and β-tubulin*** are major components of the microtubule cytoskeleton that play important roles in the polarized growth of single-celled cotton fibre^71,72^. We identified 32 putative actin-encoding genes (19 in *G.* *raimondii*) and 59 putative β-tubulin–encoding genes (20 in *G.* *raimondii*) in the *G. barbadense* genome. Phylogenetic analysis of these gene families indicated that the β-tubulin gene family showed expansion in the *G. barbadense* genome relative to the *G.* *raimondii* diploid genome. Analysis of expression profiles suggested that a large number of these genes are preferentially expressed in fibre, indicating their vital roles in cotton fibre development (**Supplementary Figure 9a,b**).

***Profilins***, a group of important actin monomer binding proteins^73,74^, are also expanded in the *G. barbadense* genome (21 in *G. barbadense* vs. 9 in *G.* *raimondii*). We observed that 13 of these were preferentially expressed at fibre initiation (during the ovule stage) (**Supplementary Figure 9c**).

***FLA*** (Fasciclin-Like Arabinogalactan proteins)^75^, a subclass of arabinogalactan proteins (AGPs), are likely involved in cotton fibre development. We noted that this gene family was conserved in each subgenome of *G. barbadense* and in the diploid *G.* *raimondii* genome. However, nearly half of the genes in this family were preferentially expressed during fibre elongation and second cell wall synthesis stages, consistent with previous observations (**Supplementary Figure 9d**).

***APX*** (Ascorbate PeroXidase) participates in the regulation of levels of intracellular reactive oxygen species (ROS) and previous research suggests that it is involved in hydrogen peroxide homeostasis during cotton fibre development^76^. We noted that this gene family was expanded in both subgenomes of *G. barbadense* (15 in At and 17 in Dt) and in the diploid *G.* *raimondii* genome (13 in D5 genome). We observed that one clade of this gene family was preferentially expressed at the fibre elongation stage (**Supplementary Figure 9e**).

***LIM***, (LIN-11, Isl1 and MEC-3 domain proteins), acts as an actin bundler to facilitate elongation of fibre cells and functions as a transcription factor involved in phenylpropanoid biosynthesis to assist in the synthesis of secondary cell walls^77^, is expanded in gene family context (33 in *G. barbadense* vs. 14 in *G.* *raimondii*). We noted that the genes belonging to class II and class III were activated during the fibre elongation and second cell wall synthesis stages (**Supplementary Figure 9f**).

# REFERENCE

1. Paterson, A. H., Brubaker, C. L., Wendel, J. F. A rapid method for extraction of cotton (*Gossypium* spp.) genomic DNA suitable for RFLP or PCR analysis. *Plant Mol. Biol. Rep.* **11**, 122–127 (1993).

2. Hillmer, A. M. *et al*. Comprehensive long-span paired-end-tag mapping reveals characteristic patterns of structural variations in epithelial cancer genomes. *Genome Res*. **21**, 665–675 (2011).

3. Yao, F. *et al.* Long Span DNA Paired-End-Tag (DNA-PET) Sequencing Strategy for the Interrogation of Genomic Structural Mutations and Fusion-Point-Guided Reconstruction of Amplicons. *PLoS ONE* **7**, e46152 (2012).

4. Martin, M. Cutadapt removes adapter sequences from high-throughput sequencing reads. *EMBnet journal*. **17**, 10–12 (2011).

5. SSearch. http://www.biology.wustl.edu/gcg/ssearch.html.

6. Ling, H. *et al*. Draft genome of the wheat A-genome progenitor Triticum urartu. *Nature* **496**, 87–90 (2013).

7. Li, R. *et al*. The sequence and de novo assembly of the giant panda genome. *Nature* **463**, 311–317 (2010).

8. Marçais, G., Kingsford, C. A fast, lock-free approach for efficient parallel counting of occurrences of k-mers. *Bioinformatics* **27**, 764–770 (2011).

9. Kelley, D., Schatz, M., Salzberg, S. Quake: quality-aware detection and correction of sequencing errors. *Genome Biol*. **11**, R116 (2010).

10. GapCloser. http://soap.genomics.org.cn/.

11. Bonfield, J. K., Whitwham, A. Gap5—editing the billion fragment sequence assembly. *Bioinformatics* **26**, 1699–1703 (2010).

12. Zerbino, D. R., Birney, E. Velvet: Algorithms for de novo short read assembly using de Bruijn graphs. *Genome Res*.**18**, 821–829 (2008).

13. VelvetOptimiser. http://bioinformatics.net.au/software.velvetoptimiser.shtml.

14. Li, H., Durbin, R. Fast and accurate short read alignment with Burrows–Wheeler transform. *Bioinformatics* **25**, 1754–1760 (2009).

15. Li, H. *et al*. The Sequence Alignment/Map format and SAMtools. *Bioinformatics* **25**, 2078–2079 (2009).

16. Picard. http://picard.sourceforge.net/.

17. Quinlan, A. R., Hall, I. M. BEDTools: a flexible suite of utilities for comparing genomic features. *Bioinformatics* **26**, 841–842 (2010).

18. Schwartz, S. *et al*. Human–Mouse Alignments with BLASTZ. *Genome Res*. **13**, 103–107 (2003).

19. Zhang, T. *et al*. Sequencing of allotetraploid cotton (Gossypium hirsutum L. acc. TM-1) provides a resource for fiber improvement. *Nat. Biotech*. **33**, 531–537 (2015).

20. Benson, G. Tandem repeats finder: a program to analyze DNA sequences. *Nucleic Acids Res*. **27**, 573–580 (1999).

21. McCarthy, E. M., McDonald, J. F. LTR_STRUC: a novel search and identification program for LTR retrotransposons. *Bioinformatics* **19**, 362–367 (2003).

22. Ellinghaus, D., Kurtz, S., Willhoeft, U. LTRharvest, an efficient and flexible software for de novo detection of LTR retrotransposons. *BMC Bioinformatics* **9**, 18 (2008).

23. Rho, M., Choi, J-H., Kim, S., Lynch, M., Tang, H. De novo identification of LTR retrotransposons in eukaryotic genomes. *BMC Genomics* **8**, 90 (2007).

24. TransposonPSI. http://transposonpsi.sourceforge.net/.

25. RepeatModeler. http://www.repeatmasker.org/RepeatModeler.html.

26. Han, Y., Wessler, S. R. MITE-Hunter: a program for discovering miniature inverted-repeat transposable elements from genomic sequences. *Nucleic Acids Res*. **38**, e199 (2010).

27. Li, W., Godzik, A. Cd-hit: a fast program for clustering and comparing large sets of protein or nucleotide sequences. *Bioinformatics* **22**, 1658–1659 (2006).

28. Repbase. http://www.girinst.org/.

29. Abrusán, G., Grundmann, N., DeMester, L., Makalowski, W. TEclass—a tool for automated classification of unknown eukaryotic transposable elements. *Bioinformatics* **25**, 1329–1330 (2009).

30. Feschotte, C., Keswani, U., Ranganathan, N., Guibotsy, M. L., Levine, D. Exploring Repetitive DNA Landscapes Using REPCLASS, a Tool That Automates the Classification of Transposable Elements in Eukaryotic Genomes. *Genome Biol Evol*. **1**, 205–220 (2009).

31. Llorens, C. *et al*. The Gypsy Database (GyDB) of mobile genetic elements: release 2.0. *Nucleic Acids Res*. **39**, D70–D74 (2011).

32. Haas, B. *et al*. Automated eukaryotic gene structure annotation using EVidenceModeler and the Program to Assemble Spliced Alignments. *Genome Biol*. **9**, R7 (2008).

33. Stanke, M. *et al*. AUGUSTUS: ab initio prediction of alternative transcripts. *Nucleic Acids Res*. **34**, W435–W439 (2006).

34. Guigó, R., Knudsen, S., Drake, N., Smith, T. Prediction of gene structure. J. Mol. Biol. 226, 141–157 (1992).

35. Majoros, W. H., Pertea, M., Salzberg, S. L. TigrScan and GlimmerHMM: two open source ab initio eukaryotic gene-finders. *Bioinformatics* **20**, 2878–2879 (2004).

36. Salamov, A. A., Solovyev, V. V. Ab initio Gene Finding in Drosophila Genomic DNA. *Genome Res*. **10**, 516–522 (2000).

37. Burge, C., Karlin, S. Prediction of complete gene structures in human genomic DNA. *J. Mol. Biol*. **268**, 78–94 (1997).

38. TAIR. http://www.arabidopsis.org/.

39. Ming, R. *et al*. The draft genome of the transgenic tropical fruit tree papaya (Carica papaya Linnaeus). *Nature* **452**, 991–996 (2008).

40. Jaillon, O. *et al*. The grapevine genome sequence suggests ancestral hexaploidization in major angiosperm phyla. *Nature* **449**, 463–467 (2007).

41. Argout, X. *et al*. The genome of Theobroma cacao. *Nat. Genet.* **43**, 101–108 (2011).

42. Tuskan, G. A. *et al*. The Genome of Black Cottonwood, Populus trichocarpa (Torr. & Gray). *Science* **313**, 1596–1604 (2006).

43. She, R., Chu, J. S-C., Wang, K., Pei, J., Chen, N. genBlastA: Enabling BLAST to identify homologous gene sequences. *Genome Res*. **19**, 143–149 (2009).

44. Birney E, Clamp M, Durbin R. GeneWise and Genomewise. *Genome Res*. **14**, 988–995 (2004).

45. Haas, B. J. *et al*. Improving the Arabidopsis genome annotation using maximal transcript alignment assemblies. *Nucleic Acids Res.* **31**, 5654–5666 (2003).

46. Trapnell, C., Pachter, L., Salzberg, S. L. TopHat: discovering splice junctions with RNA-Seq. *Bioinformatics* **25**, 1105–1111 (2009).

47. Trapnell, C. *et al*. Differential gene and transcript expression analysis of RNA-seq experiments with TopHat and Cufflinks. *Nat. Protocols.* **7**, 562–578 (2012).

48. Haas, B. *et al*. Automated eukaryotic gene structure annotation using EVidenceModeler and the Program to Assemble Spliced Alignments. *Genome Biol*. **9**, R7 (2008).

49. Zhu, L., Tu, L., Zeng, F., Liu, D., Zhang, X. An Improved Simple Protocol for Isolation of High Quality RNA from *Gossypium* spp. Suitable for cDNA Library Construction. *Acta Agron Sin.* **31**, 1657–1659 (2005).

50. FASTX-Toolkit. http://hannonlab.cshl.edu/fastx_toolkit/.

51. Wicker, T. *et al*. A unified classification system for eukaryotic transposable elements. *Nat. Rev. Genet.* **8**, 973–982 (2007).

52. Edgar, R. C. MUSCLE: multiple sequence alignment with high accuracy and high throughput. *Nucleic Acids Res.* **32**, 1792–1797 (2004).

53. Olson, S. A. Emboss opens up sequence analysis. *Brief Bioinform* **3**, 87–91 (2002).

54. Shannon, P. *et al*. Cytoscape: A Software Environment for Integrated Models of Biomolecular Interaction Networks. *Genome Res.* **13**, 2498–2504 (2003).

55. Tamura, K., Stecher, G., Peterson, D., Filipski, A., Kumar, S. MEGA6: Molecular Evolutionary Genetics Analysis Version 6.0. *Mol. Biol. Evol.* **30**, 2725–2729 (2013).

56. Vitte, C., Panaud, O. Formation of Solo-LTRs Through Unequal Homologous Recombination Counterbalances Amplifications of LTR Retrotransposons in Rice Oryza sativa L. *Mol. Biol. Evol.* **20**, 528–540 (2003).

57. Devos, K. M., Brown, J. K. M., Bennetzen, J. L. Genome Size Reduction through Illegitimate Recombination Counteracts Genome Expansion in Arabidopsis. *Genome Res.* **12**, 1075–1079 (2002).

58. Hughes, S. E., Hawley, R. S. Heterochromatin: A Rapidly Evolving Species Barrier. *PLoS Biol.* **7**, e1000233 (2009).

59. McKenna, A. *et al*. The Genome Analysis Toolkit: A MapReduce framework for analyzing next-generation DNA sequencing data. *Genome Res.* **20**, 1297–1303 (2010).

60. Ruden, D. M., Lu, X. A program for annotating and predicting the effects of single nucleotide polymorphisms, SnpEff: SNPs in the genome of Drosophila melanogaster strain w1118; iso-2; iso-3. *Fly*. **6**, 80–92 (2012).

61. Li, L., Stoeckert, C. J., Roos, D. S. OrthoMCL: Identification of Ortholog Groups for Eukaryotic Genomes. *Genome Res.* **13**, 2178–2189 (2003).

62. Paterson, A. H. *et al*. Repeated polyploidization of Gossypium genomes and the evolution of spinnable cotton fibres. *Nature* **492**, 423–427 (2012).

63. Leitch, A. R., Leitch, I. J. Genomic Plasticity and the Diversity of Polyploid Plants. *Science* **320**, 481–483 (2008).

64. Consortium TBrGSP. The genome of the mesopolyploid crop species Brassica rapa. *Nat. Genet.* **43**, 1035–1039 (2011).

65. HMMER. http://hmmer.janelia.org/.

66. Pérez-Rodríguez, P. *et al*. PlnTFDB: updated content and new features of the plant transcription factor database. *Nucleic Acids Res.* **38**, D822–D827 (2010).

67. Wang, Y. *et al*. MCScanX: a toolkit for detection and evolutionary analysis of gene synteny and collinearity. *Nucleic Acids Res.* **40**, e49 (2012).

68. Sturn, A., Quackenbush, J., Trajanoski, Z. Genesis: cluster analysis of microarray data. *Bioinformatics* **18**, 207–208 (2002).

69. Bio-pipeline. https://github.com/tanghaibao/bio-pipeline.

70. Larkin, M. A. *et al*. Clustal W and Clustal X version 2.0. *Bioinformatics* **23**, 2947–2948 (2007).

71. Li, X. B., Fan, X. P., Wang, X. L., Cai, L., Yang, W. C. The Cotton ACTIN1 Gene Is Functionally Expressed in Fibers and Participates in Fiber Elongation. *Plant Cell* **17**, 859–875 (2005).

72. Li, L., Wang, X. L., Huang, G. Q., Li, X. B. Molecular characterization of cotton GhTUA9 gene specifically expressed in fibre and involved in cell elongation. *J. Exp. Bot.* **58**, 3227–3238 (2007).

73. He, X. C., Qin, Y. M., Xu, Y., Hu, C. Y., Zhu, Y. X. Molecular cloning, expression profiling, and yeast complementation of 19 beta-tubulin cDNAs from developing cotton ovules. *J. Exp. Bot.* **59**, 2687–2695 (2008).

74. Wang, J. *et al*. Over-expression of a Profilin (GhPFN2) Promotes the Progression of Developmental Phases in Cotton Fibers. *Plant Cell Physiol.* **51**, 1276–1290 (2010).

75. Huang, G. Q. *et al*. A Fasciclin-like Arabinogalactan Protein, GhFLA1, is Involved in Fiber Initiation and Elongation of Cotton (Gossypium hirsutum). *Plant Physiol.* **161**, 1278–1290 (2013).

76. Li, H. B. *et al*. A cotton ascorbate peroxidase is involved in hydrogen peroxide homeostasis during fibre cell development. *New Phytol.* **175**, 462–471 (2007).

77. Li, Y. *et al*. A cotton LIM domain-containing protein (GhWLIM5) is involved in bundling actin filaments. *Plant Physiol. Biochem.* **66**, 34–40 (2013).
